# Chronic Disease Management to Enhance Medication Adherence Trajectories in Long‐Term Survivors of Stroke: A Population‐Based Cohort Study

**DOI:** 10.1002/pds.70148

**Published:** 2025-05-04

**Authors:** Lachlan L. Dalli, Monique F. Kilkenny, Muideen T. Olaiya, David Ung, Joosup Kim, Leonid Churilov, Dominique A. Cadilhac, Vijaya Sundararajan, Amanda G. Thrift, Mark R. Nelson, Natasha A. Lannin, Rebecca Barnden, Velandai Srikanth, Nadine E. Andrew

**Affiliations:** ^1^ Stroke and Ageing Research, Department of Medicine, School of Clinical Sciences at Monash Health Monash University Clayton Victoria Australia; ^2^ Stroke Division, Florey Institute of Neuroscience and Mental Health University of Melbourne Parkville Victoria Australia; ^3^ Department of Medicine, Peninsula Clinical School, School of Translational Medicine Monash University Frankston Victoria Australia; ^4^ National Centre for Healthy Ageing, Peninsula Clinical School School of Translational Medicine Frankston Victoria Australia; ^5^ Melbourne Medical School The University of Melbourne Melbourne Victoria Australia; ^6^ Department of Medicine, St Vincent's Hospital University of Melbourne Fitzroy Victoria Australia; ^7^ Menzies Institute for Medical Research University of Tasmania Hobart Tasmania Australia; ^8^ Department of Neuroscience, School of Translational Medicine Monash University Melbourne Victoria Australia; ^9^ Alfred Health Melbourne Victoria Australia

**Keywords:** data linkage, medication adherence, pharmacoepidemiology, primary care, stroke

## Abstract

**Purpose:**

Although chronic disease management (CDM) has been reported to improve medication adherence after stroke or transient ischaemic attack (TIA), the impact on specific patterns of medication adherence is unclear. We aimed to evaluate the population effect of receiving a CDM claim on trajectories of medication adherence in long‐term survivors of stroke/TIA.

**Methods:**

A cohort study was undertaken using observational data from PRECISE (42 Australian Stroke Clinical Registry hospitals [Victoria and Queensland; 2012–2015] linked with medication dispensing and primary care claims). Community‐dwelling adults with ≥ 1 primary care visit were included. The exposure was a CDM claim (versus no claim) in primary care within 7–18 months post‐stroke/TIA. Medication adherence (antihypertensive, antithrombotic, lipid‐lowering) was assessed between 19 and 30 months post‐stroke/TIA, using group‐based trajectory models. Average treatment effects were estimated using multi‐level logistic regression with inverse probability treatment weights.

**Results:**

Among 11 580 survivors of stroke/TIA (median age 70 years, 42% female; 45% with CDM claim), four distinct adherence patterns were identified: near‐perfect adherence, high adherence, declining adherence, and non‐use. After adjustment, having a CDM claim (vs no claim) promoted near‐perfect adherence (odds ratio [OR]: 1.16 [95% CI 1.08–1.25]) for antithrombotic medications. Whereas, having a CDM claim (vs no CDM claim) promoted high adherence for antihypertensive (OR: 1.33 [95% CI 1.24–1.44]) or lipid‐lowering (OR: 1.26 [95% CI 1.16–1.37]) medications. The odds of non‐use were also reduced by 17%–23% in those with (vs without) a CDM claim.

**Conclusions:**

CDM claims were associated with favourable trajectories of medication adherence in long‐term survivors of stroke/TIA.

**Summary**

- Incentivised delivery of chronic disease management (CDM) in primary care has been reported to enhance medication adherence following stroke. However, the population effect of CDM on specific trajectories of medication adherence is unknown.
- Among long‐term survivors of stroke or transient ischaemic attack, four distinct patterns of medication adherence were identified: near‐perfect adherence, high adherence, declining adherence, and non‐use.
- Having a primary care claim for CDM was associated with a greater odds of near‐perfect or high adherence, and a lesser odds of medication non‐use.
- Policies facilitating the delivery of CDM in primary care appear to support more favourable trajectories of medication adherence in long‐term survivors of stroke or transient ischaemic attack.

## 1 Introduction

Approximately half of all survivors of stroke experience a recurrent vascular event within 10 years [1]. Although antihypertensive, antithrombotic, and lipid‐lowering medications are strongly recommended following stroke, we have previously shown that patients are often non‐adherent [2, 3, 4]. Medication non‐adherence is associated with adverse events, including death and stroke recurrence [2, 3, 4].

Access to high‐quality, coordinated primary care is pivotal to support long‐term medication adherence following stroke. In many countries [5, 6], programmes exist to incentivise chronic disease management (CDM) in primary care. Specifically for stroke, this can provide access to integrated and multi‐disciplinary care, including comprehensive health assessment, referral to services, goal setting, and implementable actions to support self‐management [7].

In the first population‐based evaluation of a national chronic disease management programme for stroke (*n* = 11 580) [8], participants with (vs without) a CDM claim were 16%–26% more likely to be adherent to their secondary prevention medications. Similar to other trials [9, 10], medication adherence was ascertained using pharmaceutical dispensing data, and participants were deemed adherent if medications were available ≥ 80% of the time (i.e., proportion of days covered [PDC] ≥ 80%) [8]. However, this approach of arbitrarily dichotomising PDC can lead to loss of information, reduced statistical power, and masking of dynamic and nuanced fluctuations in medication adherence [4, 11, 12]. Therefore, we aimed to evaluate the population effect of having a claim for a CDM plan on trajectories of medication adherence in long‐term survivors of stroke or transient ischaemic attack (TIA).

## 2 Methods

### 2.1 Study Design

This was a secondary analysis of PRECISE [8, 13, 14]—a population‐based, comparative effectiveness study examining the population effect of a government‐funded CDM programme in primary care after stroke [15]. In the main analysis for PRECISE [8], structuring of the cohort and analytical decisions were guided by the target trial framework to minimise biases inherent in observational studies, other than a lack of randomisation [15, 16]. In the present secondary analysis, we applied a similar framework for defining our target population and target estimand (Table S1) and used landmark analysis methods to minimise immortal time bias. Briefly, the observation period was segmented into distinct exposure and outcome periods (Figure S1), and we only included those participants who survived the entire exposure period [17]. The reporting of this study follows the RECORD statement [18].

### 2.2 Eligibility

Participants were identified from the Australian Stroke Clinical Registry (AuSCR)—a nationwide registry for patients admitted with acute stroke or TIA. The AuSCR is approved to include patients using an opt‐out method of consent to maximise case ascertainment (< 3% opt‐out rate) [19]. Our cohort was restricted to adult residents of Victoria or Queensland, who were admitted to acute hospitals (*n* = 42) in these states between 2012 and 2015. The following inclusion criteria were also applied to emulate a hypothetical trial: (1) claimed ≥ 1 primary care physician visit during the exposure period (7–18 months post‐admission) to ensure all participants had an opportunity to claim a CDM plan; (2) survived the exposure period to 18 months post‐admission to avoid survivor bias; (3) remained in the community (i.e., not admitted to permanent residential aged care) as different funded programmes are available to those in permanent care; and (4) excluded those receiving palliative care as these participants would not ordinarily be recruited to a clinical trial [20].

### 2.3 Data Sources and Linkage

AuSCR participants had their data linked at the person‐level with the following government‐held administrative claims datasets: (i) Medicare Benefits Schedule (Medicare) containing transactional data for all government‐subsidised healthcare services (e.g., doctor visits, tests and limited allied health visits); (ii) Pharmaceutical Benefits Scheme (pharmaceutical dispensing) containing records of dispensed prescription medications (excluding non‐prescription, privately purchased, or other specialty‐funded medicines); (iii) the National Death Index containing information on date of death; (iv) the National Aged Care Data Clearing house containing information on admission to residential aged care; (v) Admitted and emergency department patient data (Victoria and Queensland) containing information on all inpatient discharges from all public, private, psychiatric, and repatriation hospitals and presentations to public and most private emergency departments.

### 2.4 Treatment Strategies

Since 1999, the Australian government has provided Medicare financial incentives to support the development of CDM plans in primary care [21]. As part of this programme, primary care physicians can claim an additional reimbursement (almost 50%) on top of an extended consultation fee to implement a CDM plan. Plans are expected to be developed in partnership with the patient and include written details of: healthcare needs and relevant conditions, management goals and patient action plans, treatments and services required, and a review date (recommended every 3–6 months).

Participants were classified as treated if they had ≥ 1 Medicare claim (item numbers: 229, 233, 721, 732) for a new CDM plan or a review of a previously established plan during the exposure period (7–18 months post‐admission). Participants were defined as untreated if they had no claim for a plan or review. The first 6 months post‐stroke were excluded as a washout period to avoid contamination from CDM provided in hospital or rehabilitation settings as part of discharge care planning.

### 2.5 Follow‐Up

Participants were followed from the start of the outcome period (19 months after the index event; i.e., time zero) until death, or 30 months after the index event, whichever occurred first.

### 2.6 Outcomes

The primary outcome for this sub‐study was adherence to each major therapeutic class of secondary prevention medication (antihypertensive, lipid‐lowering, and antithrombotic medications). Outcomes were measured from 19 months post‐admission (time zero) for a 1‐year period, or until death, whichever occurred first (outcome period). In the Australian Stroke Clinical Guidelines [22], it is recommended that patients be prescribed these medications at hospital discharge for long‐term use post‐discharge. While there may be valid reasons for withholding these medications (e.g., contraindications, drug–drug interactions, side effects), < 3.6% of patients in our cohort had documented contraindications in the registry to justify non‐prescription. Since antithrombotic medications are contraindicated in patients with haemorrhagic stroke [22], our analyses relating to this medication class excluded patients with intracerebral haemorrhage.

Adherence was based on the number of days with a medication supply available, measured in 30‐day intervals of the outcome period. This approach allowed for examination of more granular patterns of monthly medication adherence than the more common method of summarising overall PDC. As information on the prescribed daily dose was unavailable, we adopted a standard approach to impute typical doses using the registered product information for each medication (described in Table S2) [3, 4, 23, 24].

### 2.7 Covariates

The methods used to prepare the data, harmonise variables, and reduce missing data have been previously described in detail [8]. Briefly, we maximised the completeness of variables by imputing information from other linked datasets for the same person. Comorbidities were identified using a 5‐year lookback period in the hospital administrative data [25], and overall burden was summarised using the Charlson Comorbidity Index [26]. Regularity of primary care was examined within the 2‐year period before stroke/TIA, using an algorithm previously tested using linked AuSCR‐Medicare data [27]. The codes used to derive all study variables are provided in Table S3.

### 2.8 Statistical Analysis

#### 2.8.1 Group‐Based Trajectory Modelling

We used group‐based trajectory modelling to identify monthly patterns of adherence to each medication group over the outcome period (19–30 months) [28]. This method has been found to differentiate patterns of medication adherence with lower relative bias than summary PDC measures [29]. After estimating groups and their trajectories, each participant was assigned a probability of group membership and allocated to the group in which they had the greatest probability [28]. Using the STATA traj package [30], we sequentially tested a different combination of groups and polynomial functions to identify the best‐fitting models. The final chosen models were those with the lowest Bayesian Information Criterion, reasonably narrow confidence intervals, and excellent group discrimination based on average posterior probabilities (i.e., ≥ 70%) and odds of correct classification (i.e., ≥ 5) [31].

#### 2.8.2 Descriptive Statistics

Descriptive statistics were used to summarise the baseline characteristics (up to 6 months post‐stroke), by CDM receipt. Differences were assessed using *χ* ^2^ tests for categorical variables and Wilcoxon Rank‐sum tests for non‐parametric, continuous variables.

#### 2.8.3 Propensity Score Adjustment

In the absence of randomisation, we used Inverse Probability of Treatment Weights (IPTWs) to adjust for measured confounding between those exposed and unexposed to CDM plans. This allowed estimation of the average treatment effect, i.e., the hypothetical effect if every eligible patient were offered the treatment. We accounted for all potential variables influencing the exposure (receipt of CDM plans at 7–18 months poststroke/TIA) and outcome (medication adherence from 19 to 30 months poststroke/TIA) within the causal pathway (illustrated in Figure S2). Since we were interested in evaluating the total effect of having a claim for CDM on medication trajectories, we did not adjust for potential mediators that arose during the exposure period (i.e., we adjusted for baseline confounders up to 6 months post‐stroke only).

A logistic regression model was used to derive the propensity score for each participant based on their conditional probability of having a CDM claim. Stabilised weights were generated for participants based on the reciprocal of the probability of receiving the treatment that was actually received (i.e., 1/PS for adherent participants and 1/[1‐PS] for non‐adherent participants) [32]. Absolute standardised differences < 0.1 were ensured between treated and untreated participants for all covariates [32].

#### 2.8.4 Outcome Analysis

We compared the probability of each adherence trajectory group by receipt of a CDM claim. We fitted separate mixed effects logistic regression models to estimate the odds of each adherence trajectory compared with non‐use (reference group). Models were multi‐level with a cluster‐specific random intercept for health service regions to account for within‐cluster homogeneity in outcomes.

#### 2.8.5 Subgroup Analysis

To better interpret the results, we replicated the analysis in the following sub‐groups: women (vs men), age < 75 years (vs 75+ years), patients with stroke (vs TIA), new (vs prevalent) users of medication post‐stroke, new (vs previous) users of CDM plans, and those with irregular vs regular primary care [27]. For each sub‐group, we tested the statistical significance of the effect modifier using exposure × sub‐group product terms. Additional analyses were also undertaken to evaluate the specific timing of the CDM claim during the exposure period on adherence trajectories. A two‐tailed *p* value ≤ 0.05 was considered statistically significant. All analyses were undertaken using Stata/MP 18.0 for Windows (StataCorp, College Station, TX, USA, 2023).

## 3 Results

**FIGURE 1:**
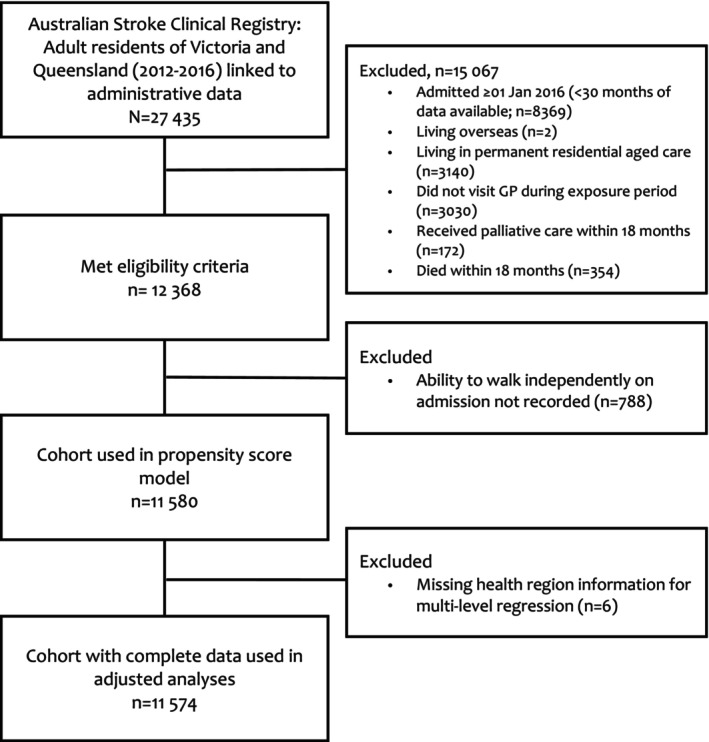
Cohort selection.

Among 11 580 participants (Figure 1), 42% were female and the median age was 70.2 years (interquartile range: 59.9–78.7). Most participants had experienced an ischaemic stroke (63%), followed by TIA (27%), intracerebral haemorrhage (8%), and undetermined stroke (3%). Overall, 45% had a CDM claim in the exposure period, between 7 and 18 months post‐admission. Approximately half (47%) of the exposed participants also had evidence of a prior CDM claim in the washout period during the first 6 months post‐admission. After applying IPTWs, the propensity score density plots had substantial overlap and covariates were well balanced between groups (Figure S3 and Table 1).

**TABLE 1:** Patient characteristics before and after matching, by receipt of a claim for chronic disease management.

	Before IPTW			After IPTW		
	No CDMP	CDMP	Std. diff.	No CDMP	CDMP	Std. diff.
	*N* = 6357	*N* = 5223		*N* = 6357	*N* = 5223	
	*n* (%)	*n* (%)		*n* (%)	*n* (%)	
At admission						
Mean age in years (SD)	66.4 (14.4)	70.7 (12.2)	0.32^a^	68.4 (13.8)	68.7 (13.1)	0.02
Female	2537 (39.9)	2337 (44.7)	0.10^a^	2703 (42.3)	2201 (42.4)	< 0.01
Married	4210 (66.2)	3479 (66.6)	0.01	4243 (66.4)	3445 (66.4)	< 0.01
Socioeconomic position						
Most disadvantaged	1270 (20.0)	1023 (19.6)	0.06	1267 (19.8)	1024 (19.7)	< 0.01
Second most disadvantaged	1041 (16.4)	927 (17.7)		1085 (17.0)	884 (17.0)	
Third most disadvantaged	1313 (20.7)	1197 (22.9)		1381 (21.6)	1123 (21.6)	
Fourth most disadvantaged	1393 (21.9)	1219 (23.3)		1445 (22.6)	1184 (22.8)	
Least disadvantaged	1340 (21.1)	857 (16.4)		1215 (19.0)	973 (18.8)	
State of residence						
Queensland	3450 (54.3)	2931 (56.1)	0.04	3513 (55.0)	2850 (54.9)	< 0.01
Victoria	2907 (45.7)	2292 (43.9)		2880 (45.0)	2338 (45.1)	
Metropolitan residence	4275 (67.2)	3520 (67.4)	< 0.01	4310 (67.4)	3484 (67.2)	0.01
Private health insurance	2520 (39.6)	1988 (38.1)	0.03	2467 (38.6)	1989 (38.3)	< 0.01
Received concessional medication benefits	3858 (60.7)	4048 (77.5)	0.37^a^	4369 (68.3)	3576 (68.9)	0.01
Type of stroke						
Intracerebral haemorrhage	479 (7.5)	398 (7.6)	0.01	501 (7.8)	417 (8.0)	0.01
Ischaemic	3964 (62.4)	3277 (62.7)		4010 (62.7)	3257 (62.8)	
TIA	1741 (27.4)	1410 (27.0)		1711 (26.8)	1377 (26.5)	
Undetermined	173 (2.7)	138 (2.6)		171 (2.7)	138 (2.7)	
Previous stroke	1052 (16.5)	1068 (20.4)	0.10^a^	1175 (18.4)	960 (18.5)	< 0.01
Unable to walk on admission	2779 (43.7)	2413 (46.2)	0.05	2887 (45.2)	2353 (45.3)	< 0.01
Interpreter needed	201 (3.2)	203 (3.9)	0.04	231 (3.6)	188 (3.6)	< 0.01
Stroke occurred while hospitalised for another condition	175 (2.8)	191 (3.7)	0.05	204 (3.2)	171 (3.3)	0.01
Treated in a stroke unit	5161 (81.2)	4309 (82.5)	0.03	5237 (81.9)	4258 (82.1)	< 0.01
Received in‐patient rehabilitation	1687 (26.5)	1732 (33.2)	0.14^a^	1931 (30.2)	1593 (30.7)	0.01
Mean Charlson Comorbidity Index (SD)	1.5 (1.8)	1.9 (1.8)	0.17^a^	1.7 (1.8)	1.7 (1.7)	0.01
Mean pre‐stroke PDC in % (SD)^b^						
Antihypertensive	40.2 (43.0)	53.9 (43.0)	0.32^a^	46.2 (43.7)	47.1 (43.6)	0.02
Antithrombotic	15.3 (32.9)	22.9 (38.5)	0.21^a^	18.3 (35.4)	19.4 (36.3)	0.03
Lipid‐lowering	29.8 (40.7)	43.9 (43.5)	0.33^a^	35.6 (42.4)	36.9 (42.7)	0.03
Comorbidities						
Angina	857 (13.5)	975 (18.7)	0.14^a^	1014 (15.9)	837 (16.1)	0.01
Atrial fibrillation	1247 (19.6)	1236 (23.7)	0.10^a^	1379 (21.6)	1128 (21.7)	< 0.01
Any cancer (excl. skin)	397 (6.2)	360 (6.9)	0.03	421 (6.6)	341 (6.6)	< 0.01
Chronic respiratory disease	307 (4.8)	376 (7.2)	0.10^a^	380 (5.9)	307 (5.9)	< 0.01
Dementia	99 (1.6)	94 (1.8)	0.02	104 (1.6)	84 (1.6)	< 0.01
Diabetes	1100 (17.3)	1737 (33.3)	0.37^a^	1582 (24.7)	1295 (25.0)	0.01
Congestive heart failure	424 (6.7)	484 (9.3)	0.10^a^	509 (8.0)	413 (8.0)	< 0.01
Myocardial infarction	448 (7.0)	466 (8.9)	0.07	513 (8.0)	421 (8.1)	< 0.01
Peripheral vascular disease	279 (4.4)	298 (5.7)	0.06	314 (4.9)	258 (5.0)	< 0.01
Dyslipidaemia	3176 (50.0)	3378 (64.7)	0.30^a^	3616 (56.6)	2949 (56.8)	0.01
Hypertension	3839 (60.4)	3832 (73.4)	0.28^a^	4253 (66.5)	3484 (67.2)	0.01
Anxiety or depression	548 (8.6)	513 (9.8)	0.04	594 (9.3)	481 (9.3)	< 0.01
Liver disease	128 (2.0)	104 (2.0)	< 0.01	129 (2.0)	108 (2.1)	< 0.01
Renal disease	464 (7.3)	565 (10.8)	0.12^a^	566 (8.9)	464 (9.0)	< 0.01
Obesity	170 (2.7)	190 (3.6)	0.06	196 (3.1)	158 (3.1)	< 0.01
Smoking history	3483 (54.8)	2905 (55.6)	0.02	3524 (55.1)	2865 (55.2)	< 0.01
Alcohol misuse	314 (4.9)	224 (4.3)	0.03	301 (4.7)	247 (4.8)	< 0.01
Carotid stenosis	349 (5.5)	325 (6.2)	0.03	381 (6.0)	298 (5.7)	0.01
Previous fracture	871 (13.7)	868 (16.6)	0.08	923 (14.4)	804 (15.5)	0.03
Previous fall	564 (8.9)	666 (12.8)	0.13^a^	637 (10.0)	602 (11.6)	0.05
Low blood pressure	766 (12.0)	813 (15.6)	0.10^a^	852 (13.3)	726 (14.0)	0.02
Coronary artery disease	562 (8.8)	609 (11.7)	0.09	668 (10.4)	515 (9.9)	0.02
Epilepsy	215 (3.4)	179 (3.4)	0.01	227 (3.6)	178 (3.4)	0.01
Up to 6 months post‐admission						
Specialist visit with						
Cardiologist	2103 (33.1)	1762 (33.7)	0.01	2129 (33.3)	1730 (33.3)	< 0.01
Geriatrician	396 (6.2)	345 (6.6)	0.02	424 (6.6)	319 (6.2)	0.02
Neurologist	2157 (33.9)	1669 (32.0)	0.04	2104 (32.9)	1702 (32.8)	< 0.01
Rehabilitation physician	633 (10.0)	600 (11.5)	0.05	691 (10.8)	571 (11.0)	< 0.01
Dispensed medications ≥ 2 times						
Antihypertensive	4920 (77.4)	4509 (86.3)	0.23^a^	5213 (81.6)	4263 (82.2)	0.02
Lipid‐lowering	5261 (82.8)	4605 (88.2)	0.15^a^	5447 (85.2)	4435 (85.5)	0.01
Antithrombotic	4964 (78.1)	4365 (83.6)	0.14^a^	5151 (80.6)	4187 (80.7)	< 0.01

Abbreviations: CDMP denotes chronic disease management claim; IPTW, inverse probability treatment weight; PDC, proportion of days covered; SD, standard deviation; Std. diff, absolute standardised difference; TIA, transient ischaemic attack.

^a^ Absolute standardised difference ≥ 0.1, signifying imbalance between groups.

^b^ During the 1‐year period before admission.

**FIGURE 2:**
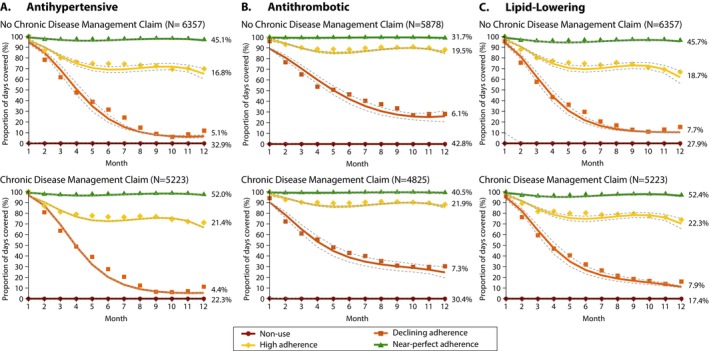
Group‐based trajectory models outlining patterns of medication use in long‐term survivors of stroke or transient ischaemic attack, by medication group and receipt of a claim for chronic disease management. For each plot, solid lines represent the predicted probabilities of group membership; dashed grey lines represent the corresponding 95% confidence intervals; and symbols represent the actual adherence in each group weighted on the posterior probability of group membership. Percentages shown beside each line represent the proportion of patients assigned to each trajectory group.

During the outcome period (19 and 30 months post‐discharge), 8948 (72.4%) patients were dispensed an antihypertensive, 7244 (63.5%) were dispensed an antithrombotic, and 9564 (77.3%) were dispensed a lipid‐lowering medication. After undertaking group‐based trajectory modelling (Table S4) and accounting for deaths in 516 (4.2%) participants during the follow‐up period, four distinct patterns of medication adherence were identified (Figure 2 and Table S5). Based on the shapes of these trajectories, Trajectory 1 was defined as non‐use; Trajectory 2, declining adherence; Trajectory 3, high adherence; and Trajectory 4, near‐perfect adherence.

Participants with more favourable medication adherence trajectories (i.e., near‐perfect or high vs. non‐use or declining) were slightly older, had a greater prevalence of specific comorbidities (e.g., hypertension, dyslipidaemia), and visited their primary care physician more regularly (Table S6). In contrast, non‐users of medication were more likely to have experienced a TIA (vs stroke) and were less likely to have been treated in a stroke unit. The proportion of patients with high or near‐perfect adherence was greater in those with versus without a CDM claim (antihypertensive: 73% vs. 62%; antithrombotic: 62% vs. 51%; lipid‐lowering: 75% vs. 64%).

In unadjusted analyses (Table 2), having a CDM claim was associated with reduced odds of non‐use of each medication and increased odds of a high or near‐perfect adherence. After IPTW adjustment (Table 2), the magnitude of the ORs were substantially attenuated. Patients with (vs without) a CDM claim were more likely to have near‐perfect adherence for antithrombotic medications (OR: 1.16 [95% CI: 1.08–1.25]). However, for lipid‐lowering and antihypertensive medications, CDM claims promoted high adherence only (lipid‐lowering: OR: 1.26 [95% CI: 1.16–1.37]; antihypertensive: OR: 1.33 [95% CI: 1.24–1.44]). Across all medications following adjustment, receipt of a CDM claim reduced the odds of medication non‐use but had no observable effect on declining adherence.

**TABLE 2:** Effect of claiming a chronic disease management plan on medication adherence trajectories in long‐term survivors of stroke or transient ischaemic attack, by medication group.

	Trajectory 1: Non use		Trajectory 2: Declining adherence		Trajectory 3: High adherence		Trajectory 4: Near‐perfect adherence	
	Unadjusted	Adjusted^a^	Unadjusted	Adjusted^a^	Unadjusted	Adjusted^a^	Unadjusted	Adjusted^a^
	OR (95% CI)	OR (95% CI)	OR (95% CI)	OR (95% CI)	OR (95% CI)	OR (95% CI)	OR (95% CI)	OR (95% CI)
Antihypertensive *N* = 11 580	0.58 (0.53–0.63)	0.83 (0.77–0.90)	0.85 (0.72–1.01)	0.84 (0.65–1.08)	1.34 (1.22–1.47)	1.33 (1.24–1.44)	1.33 (1.24–1.43)	1.00 (0.93–1.07)
Antithrombotic *N* = 10 703^b^	0.58 (0.53–0.63)	0.81 (0.76–0.86)	1.20 (1.03–1.40)	1.17 (0.93–1.47)	1.16 (1.06–1.28)	1.03 (0.96–1.11)	1.48 (1.37–1.61)	1.16 (1.08–1.25)
Lipid‐lowering *N* = 11 580	0.54 (0.49–0.59)	0.77 (0.70–0.84)	1.03 (0.89–1.18)	1.07 (0.92–1.25)	1.25 (1.14–1.36)	1.26 (1.16–1.37)	1.32 (1.33–1.42)	1.01 (0.94–1.08)

Abbreviations: CI, confidence interval; OR, odds ratio; Pint, interaction *p* value; TIA, transient ischaemic attack.

^a^ Derived using multi‐level logistic regression, adjusted using inverse probability treatment weights.

^b^ Excludes participants with intracerebral haemorrhage.

There was minimal evidence of effect modification across the examined sub‐groups, based on *p* for interaction ≤ 0.05 (Tables S7–S9). For non‐use of antithrombotic medications, effects were greater in those with stroke vs. TIA. For non‐use of lipid‐lowering medications, effects were attenuated in older (75+ years) versus younger (< 75 years) participants, and in those who newly received a CDM claim versus previous CDM recipients. A significant interaction was observed by sex, whereby CDM claims appeared to only increase the odds of high adherence to antithrombotic medications in women, but not men. For antithrombotic medications, we also found evidence of effect modification by the regularity of primary care, with greater effect sizes observed in those with more regular (vs less regular) primary care. Results were similar when we considered the timing of the CDM claim during the exposure period (Table S10). Only the association for non‐use of lipid‐lowering medications was diminished for participants who received a CDM claim in the final quarter of the exposure period (vs earlier quarters).

## 4 Discussion

In this study, we provide real‐world evidence on the population‐level effect of a national CDM programme on trajectories of medication adherence among long‐term survivors of stroke/TIA. Specifically, having a CDM claim between 6 and 18 months post‐stroke promoted high adherence to lipid‐lowering and antihypertensive medications, and near‐perfect adherence for antithrombotic medications, in the subsequent year. Importantly, those with (vs without) a CDM claim were less likely to be non‐users of each medication. In line with the intended use of these plans, our findings suggest that national programmes to incentivise CDM in long‐term survivors of stroke not only increase the uptake of secondary prevention medications but also promote sustained medication adherence.

These results support the main outcome analysis of PRECISE [8], providing additional compelling evidence to support population‐funded programmes for CDM in primary care. In the primary PRECISE study [8], CDM claims were shown to positively increase the odds of being adherent (i.e., PDC ≥ 80%) by 16%–26%. Our use of group‐based trajectory modelling in the present study provides a more nuanced understanding of the population‐level effect of CDM policies on distinct patterns of medication use. It is likely that current results are mediated by more comprehensive cardiovascular risk assessment and medication counselling in CDM recipients, which in turn facilitated ongoing medication adherence, compared with the usual care group [7].

The shape of trajectories in our study is similar to previous studies in patients with hypertension and atrial fibrillation [33, 34, 35]. Interestingly, the trajectories in our study were relatively similar between those with and without a CDM claim. However, the proportional membership differed, with more favourable trajectories observed in those with (vs without) a CDM claim. Although we were unable to detect an effect of CDM claims on declining adherence, this group had the smallest number of participants and may have been under‐powered to detect a difference. Further research is needed to understand reasons underlying the observed rapid decline in medication adherence at approximately 6 months during our outcome period (or 24 months post‐stroke). Potentially, this may have been driven by appropriate clinical management (e.g., due to side effects or attainment of risk factor targets), patient preferences, social factors (e.g., cost), or cessation of an existing CDM plan.

Our results concord with a previous Korean study in patients with type 2 diabetes (2010–2013; *N* = 2506) [36], whereby having a CDM claim was associated with a 6% increase in 1‐year adherence to antihypertensive medications. The authors found greater effects in patients who visited the same (versus different) physicians. This finding is similar to the sub‐group analysis in the present study, highlighting the importance of maintaining regular and continuous care with the same primary care physician to optimise the effectiveness of CDM plans on medication adherence.

Prior to this study, there was limited evidence on the potential effect of CDM policies on medication adherence in cardiovascular disease, with most studies being cross‐sectional or relying on self‐reported measures of adherence [37, 38]. Other strengths of this study include the large sample of long‐term survivors of stroke or TIA identified by clinicians in acute hospitals participating in the AuSCR. We adopted a range of techniques to minimise bias in the outcome analysis, including incorporation of aspects of the target trial framework, group‐based trajectory models, landmark analysis, and IPTW adjustment. Linkage with pharmaceutical claims data enabled more objective assessment of medication adherence than self‐reported measures.

Limitations include the lack of data on medications supplied over‐the‐counter without a prescription (i.e., aspirin) and the inability to confirm whether dispensed medications were actually taken. The lack of longitudinal prescription data meant we were unable to discern the impact of CDM claims on primary non‐adherence. Our patient cohort was limited to two Australian states due to data availability. However, these states had high participation and case ascertainment in the AuSCR during the study period, which likely minimised potential selection bias. We were also unable to discern the content of the CDM plans or the extent to which they were followed by patients. Although excellent balance was achieved for measured covariates, we cannot discount the possibility of residual and unmeasured confounding, particularly related to positive health‐seeking behaviours associated with the use of CDM plans and preventive medications. While validated coding algorithms were applied where possible to ascertain confounders, there remains potential for misclassification bias if certain medical information was omitted or incorrectly coded. Lastly, our landmarking approach helped to overcome immortal time bias by partitioning the data into discrete exposure and outcome periods. However, this approach may have introduced selection bias to favour healthier individuals who survived to 18 months after a stroke or TIA. Hence, our findings may only be generalisable to long‐term survivors of stroke or TIA who are in the care of the primary care physician at 18 months post‐admission.

### 4.1 Conclusions

Among long‐term survivors of stroke or TIA, access to government‐funded CDM in primary care promoted population‐level improvements in high and near‐perfect medication adherence, and reduced medication non‐use. National programmes to incentivise the delivery of CDM in primary care should be encouraged to maximise long‐term adherence to secondary prevention medications after stroke or TIA.

### 4.2 Plain Language Summary

Stroke is a chronic disease which often requires complex management within primary care and prescription of multiple cardiovascular medicines. Within Australia and many other universal healthcare systems, chronic disease management (CDM) programmes have been introduced within primary care to provide more comprehensive risk factor assessment and subsidised access to healthcare services. However, the impact of these CDM plans on trajectories of medication adherence in long‐term survivors of stroke is unclear. In this study, we evaluated whether trajectories of medication adherence differed in long‐term survivors of stroke based on whether a claim for CDM was made. This analysis was undertaken using data from a national stroke registry linked with prescription dispensing records. After accounting for differences in patient characteristics, patients with a CDM claim had more favourable long‐term trajectories of medication adherence than those without a CDM claim. Specifically, those with a CDM claim were more likely to have high or near‐perfect adherence, and less likely to be a non‐user of medication. These findings suggest that CDM programmes in primary care appear to be beneficial for supporting medication adherence in long‐term survivors of stroke.

## Author Contributions

L.L.D. was responsible for formal analysis, investigation, and writing the original draft. N.E.A. and M.F.K. were responsible for supervision. N.E.A., M.F.K., and J.K. were responsible for funding acquisition. All authors contributed to conceptualisation, methodology, and reviewing and editing the manuscript for intellectual content. All authors approved the final version for submission. The corresponding author certifies that all mentioned authors satisfy the requirements for authorship.

## Disclosure

Funders played no role in the design of the study, data collection, analysis, and interpretation of data, decision to publish, and in the writing of the manuscript.

## Ethics Statement

This study was conducted in accordance with the Declaration of Helsinki 1964, as revised in 2008. Approval was obtained from Human Research Ethics committees at Monash University (Approval: #12301) and the Australian Institute of Health and Welfare (Approval #EO2018/2/449). Approvals for linkage and release of de‐identified data were obtained from relevant data custodians, including the Australian Stroke Clinical Registry (AuSCR) and the Australian Department of Health.

## Consent

The authors have nothing to report.

## Conflicts of Interest

D.A.C. is the current Data Custodian for the AuSCR and Executive Officer of the Australian and New Zealand Stroke Organisation (formerly the Stroke Society of Australasia). D.A.C., and M.F.K. are members of the AuSCR Steering or Management Committees. A.G.T. is a previous Board Member of the Stroke Foundation; M.F.K. is a member of the Research Advisory Committee of the Stroke Foundation. D.A.C. reports receiving restricted grants from Amazon Web Services, Boehringer Ingelheim, and Moleac outside the submitted work. M.R.N. reports membership of a Novartis lipids advisory board outside the submitted work. All other authors report no conflicts.

## Supporting information


**Data S1.** Supplementary Information.

## Data Availability

Due to ethical and legal restrictions, person‐level data from this study cannot be shared. However, certain aggregated data and coding that support the findings of this study are available on reasonable request from the corresponding author, following approval from the relevant data custodians.
