# Supplementary material for: Chronic Disease Management to Enhance Medication Adherence Trajectories in Long‐Term Survivors of Stroke: A Population‐Based Cohort Study
Source: Pharmacoepidemiol Drug Saf. 2025 May 4;34(5):e70148. doi: 10.1002/pds.70148 (PMC12050132; doi:10.1002/pds.70148)
Supplement: Supplementary file 1 — Data S1. Supplementary Information. [file PDS-34-e70148-s001.docx]

**Supplemental Material**

**Chronic Disease Management to Enhance Medication Adherence Trajectories in Long-Term Survivors of Stroke:**

**A Population-Based Cohort Study**

Lachlan L. Dalli, Monique F. Kilkenny, Muideen T. Olaiya, David Ung, Joosup Kim,
Leonid Churilov, Dominique A. Cadilhac, Vijaya Sundararajan, Amanda G. Thrift,
Mark R. Nelson, Natasha A. Lannin, Rebecca Barnden, Velandai Srikanth,

Nadine E. Andrew

**Supplemental Tables**

**Table S1.** Study features

**Table S2.** Operationalisation of medication adherence parameters based on the ten-spiders tool

**Table S3.** Datasets and codes used to derive study variables

**Table S4**. Group-based trajectory model iterations, by medication group and trajectory shapes

**Table S5**. Median proportion of days covered, by medication adherence trajectory and chronic disease management claim

**Table S6**. Participant characteristics, by medication and adherence trajectory

**Table S7**. Effect of having a claim for chronic disease management on trajectories of antihypertensive adherence, overall and by sub-groups

**Table S8**. Effect of having a claim for chronic disease management on trajectories of antithrombotic adherence, overall and by sub-groups

**Table S9**. Effect of having a claim for chronic disease management on trajectories of lipid-lowering adherence, overall and by sub-groups

**Table S10.** Association of having a chronic disease management claim on trajectories of medication adherence, by medication group and timing of chronic disease management claim during the exposure period

**supplemental figures**

**Figure S1.** Schematic of study design

**Figure S2.** Directed acyclic graph of hypothesised causal pathway

**Figure S3**. Density plots of propensity scores for participants with and without a chronic disease management claim, before and after inverse probability treatment weighting

**Appendices**

**Appendix S1.** Co-investigators and other contributors to the Australian Stroke Clinical Registry

**Table S1. Study features**

| **Feature** | **Operationalisation** |
| --- | --- |
| **Eligibility Criteria** | ***Sample selection:***  Adults living in the community with at least one claim for a primary care physician visit between 7 and 18 months post-admission for stroke or transient ischaemic attack to ensure opportunity to receive the intervention. To align eligibility with traditional RCTs, those receiving palliative care or admitted to residential care were also excluded. |
| **Treatment strategies** | ***Exposure group:***  Had a Medicare claim for a CDM plan in the 7-18 months post-admission for stroke or transient ischaemic attack  ***Comparator group:***  Did not have a claim for a Medicare CDM plan in the 7-18 months post-admission for stroke or transient ischaemic attack |
| **Assignment Procedures** | Inverse probability treatment weighting based on >45 measured confounders up to 6 months post-admission for stroke or transient ischaemic attack |
| **Follow-up** | Follow-up starts at 19 months post-stroke (t_0_) for a 1-year period until 30 months post stroke, or death, whichever occurred first. |
| **Outcome(s)** | Medication adherence measured in monthly intervals using prescription refill claims data. |
| **Causal contrast(s)** | Intention to treat for those with at least one CDM plan or review.  Sensitivity analysis for new users of a CDM plan. |

CDM denotes chronic disease management; and RCT, randomised controlled trial.

**Table S2. Operationalisation of medication adherence parameters based on the ten-spiders tool**

| **PDC parameter** | **Operationalisation** |
| --- | --- |
| **T – Threshold** | To more precisely investigate medication adherence, PDC was analysed as a continuous variable (i.e., number of days with access to a medication supply within each 30-day window of the denominator period). |
| **E – Eligibility** | All community-dwelling survivors of stroke who survived the exposure period (i.e., to 18 months post-stroke). |
| **N – Numerator and denominator** | **PDC numerator**: Total number of days with medication available during each 30-day window from the first date of medication dispensing, between 19 and 30 months post-stroke.  **PDC denominator**: Twelve 30-day windows, between 19 and 30 months post-stroke. |
| **S – Survival** | Follow-up ended at the date of death for participants who died between 19 and 30 months post-stroke (i.e. the PDC numerator and denominator were censored at the date of death). |
| **P – Pre-Supply** | A 3-month lookback period (i.e. between 15 and 18 months post-stroke) was used to identify previous supplies of medication. Any unused supply at the beginning of the start of follow-up contributed to the PDC numerator. |
| **I – In-hospital supplies** | Information was unavailable on medications supplied in hospital.  Therefore, hospitalised days contributed to the PDC numerator as it was assumed that separate (in-patient) medication supplies would be provided to patients who were admitted to hospital. |
| **D – Doses** | Information on the prescribed daily dose was unavailable. Consequently, standard doses were imputed using the registered product information for each medication. In brief, all medicines were assigned a dose of one per day, apart from the following medicines with twice daily dosing: captopril, dabigatran, apixaban, dipyridamole, ticagrelor, dipyridamole and aspirin, nifedipine, verapamil, frusemide, and gemfibrozil. |
| **ER – Early Refills** | The numerator was extended to account for early refills of the same medication (i.e. carry-over granted). |
| **S – Switching** | If patients switched between different antihypertensive medications within the same drug class (e.g. enalapril to perindopril), we assumed the initial medication was discontinued once the new medication was supplied (i.e. carry-over not granted). |

PDC denotes proportion of days covered.

The TEN-SPIDERS tool is a reporting checklist to improve the transparent and systematic reporting of PDC and its parameters^1^

Adapted from Andrew NE et al. *Lancet Reg Health West Pac*. 2023;34:100723 (<https://doi.org/10.1016/j.lanwpc.2023.100723>).

1. DALLI LL, Kilkenny MF, Arnet I, Sanfilippo FM, Cummings DM, Kapral MK, Kim J, Cameron J, Yap KY, Greenland M, et al. Towards better reporting of the proportion of days covered method in cardiovascular medication adherence: A scoping review and new tool TEN-SPIDERS. *Br. J. Clin. Pharmacol*. 2022;88:4427-4442. doi: 10.1111/bcp.15391

**Table S3. Datasets and codes used to derive study variables**

| **Variable** | **Dataset** | **Relevant codes** |
| --- | --- | --- |
| **Exposure variable:** |  |  |
| Chronic disease management plan claim | Medicare Benefits Schedule | Item codes: 00229, 00233, 00721, 00732 |
| **Outcome variable:** |  |  |
| Antihypertensive medications | Pharmaceutical Benefits Scheme | ATC codes: C08, C09, C03AA03, C03BA04, C03BA11, C10BX03 |
| Lipid-lowering medications | Pharmaceutical Benefits Scheme | ATC codes: C10 |
| Antithrombotic medications | Pharmaceutical Benefits Scheme | ATC codes: B01 (excluding B01AC06) |
| **Covariates:** |  |  |
| Age | AuSCR | Not applicable (collected in registry) |
| Female | AuSCR | Not applicable (collected in registry) |
| Married | Hospital administrative | At least one hospitalisation coded with the marital status of ‘Married/de facto’ in the 1.5-year period before the exposure period |
| Socioeconomic position | IRSAD | Linked to residential postcodes of participants in the AuSCR |
| State of residence | AuSCR | Not applicable (collected in registry) |
| Metropolitan residence | Australian Statistical Geography Standard | Linked to residential postcodes of participants in the AuSCR |
| Private health insurance | Hospital administrative | At least one hospitalisation funded by private health insurance in the 1.5-year period before the exposure period |
| Received concessional medication benefits | Pharmaceutical Benefits Scheme | At least 90% of medicines dispensed with a concession card subsidy in the 2.5-year period before the outcome period |
| Type of stroke | AuSCR | Not applicable (collected in registry) |
| Undetermined | AuSCR | Not applicable (collected in registry) |
| Previous stroke | AuSCR | Not applicable (collected in registry) |
| Unable to walk on admission | AuSCR | Not applicable (collected in registry) |
| Interpreter needed | AuSCR | Not applicable (collected in registry) |
| Stroke occurred while hospitalised for another condition | AuSCR | Not applicable (collected in registry) |
| Treated in a stroke unit | AuSCR | Not applicable (collected in registry) |
| Received in-patient rehabilitation | Hospital administrative | ICD-10 codes: Z466-Z469, Z501, Z504, Z505, Z507-Z509, Z602, Z608, Z609, Z764, Z765, Z768, Z769 |
| Angina | Hospital administrative | ICD-10 codes: I20, I240, I248, I249 |
| Atrial fibrillation | Hospital administrative | ICD-10 codes: I48 |
| Any cancer (excl. skin) | Hospital administrative | ICD-10 codes: C00, C1, C21-C26, C30-C34, C37-C41, C43, C45- C58, C60-C72, C74-C76, C81-C85, C880, C882, C883, C887, C889, C900, C901, C91-C93, C940, C941, C942, C943, C944, C945, C947, C95-C97 |
| Chronic respiratory disease | Hospital administrative | ICD-10 codes: I278, I279, J40, J41, J42, J43, J44, J45, J46, J47, J60, J61, J62, J63, J64, J65, J66, J67, J684, J701, J703 |
| Dementia | Hospital administrative | ICD-10 codes: F00, F01, F02, F03, F051, G30, G311 |
| Diabetes | Hospital administrative | ICD-10 codes: E10, E11, E12, E13, E14 |
| Congestive heart failure | Hospital administrative | ICD-10 codes: I099, I110, I130, I132, I255, I420, I425, I426, I427, I428, I429, I43, I50, P290 |
| Myocardial infarction | Hospital administrative | ICD-10 codes: I21, I22, I252 |
| Peripheral vascular disease | Hospital administrative | ICD-10 codes: I70, I71, I731, I738, I739, 1771, 1790, I792, K551, K558, K559, Z958, Z959 |
| Dyslipidaemia | Hospital administrative | ICD-10 codes: E78 |
| Hypertension | Hospital administrative | ICD-10 codes: I10, I11, I12, I13, I15 |
| Anxiety or depression | Hospital administrative | ICD-10 codes: F063, F064, F065, F066, F067, F068, F069, F051, F32, F33, F34, F38, F39, F41, F42, F43, F99 |
| Liver disease | Hospital administrative | ICD-10 codes: B18, K70, K71, K72, K73, K74, K75, K76, K77, Z944 |
| Renal disease | Hospital administrative | ICD-10 codes: I120, I131, N032, N033 N034, N035, N036, N037, N052, N053, N054, N055, N056, N057, N18, N19, N250, Z490, Z491, Z492, Z992 |
| Obesity | Hospital administrative | ICD-10 codes: E66 |
| Smoking history | Hospital administrative | ICD-10 codes: Z720, F17, T652, Z864 |
| Alcohol misuse | Hospital administrative | ICD-10 codes: F10, E52, G621, I426, K292, K700, K703, K709, T51, Z502, Z714, Z721 |
| Carotid stenosis | Hospital administrative | ICD-10 codes: I652, G451, S150 |
| Previous fracture | Hospital administrative | ICD-10 codes: S02, S12, S22, S32, S42, S52, S62, S72, S82, S92, M80 |
| Previous fall | Hospital administrative | ICD-10 codes: W00, W01, W02, W03, W04, W05, W06, W07, W08, W09, W10, W11, W12, W13, W14, W15, W16, W17, W18, W19 |
| Low blood pressure | Hospital administrative | ICD-10 codes: I95 |
| Coronary artery disease | Hospital administrative | ICD-10 codes: I200, I250, I251 |
| Epilepsy | Hospital administrative | ICD-10 codes: G40, G41, O15, R56 |
| Cardiologist visit | Medicare Benefits Schedule | Provider specialty codes: 004, 084 |
| Geriatrician visit | Medicare Benefits Schedule | Provider specialty codes: 616, 096  Item codes: 00141, 00143, 00145, 00147, 00149 |
| Neurologist visit | Medicare Benefits Schedule | Provider specialty codes: 009, 089 |
| Rehabilitation physician visit | Medicare Benefits Schedule | Provider specialty codes: 012, 058, 092, 412 |

AuSCR: Australian Stroke Clinical Registry; ATC: Anatomical Therapeutic Chemical Code; ICD-10: International Statistical Classification of Diseases and Related Health Problems 10th Revision (Australian Modification); IRSAD: Index of Relative Socioeconomic Advantage and Disadvantage.

**Table S4. Group-based trajectory model iterations, by medication group and trajectory shapes**

| **No. of groups** | **Trajectory shape*** | **BIC** | **Group Membership** | | | | | | | | | | | |
| --- | --- | --- | --- | --- | --- | --- | --- | --- | --- | --- | --- | --- | --- | --- |
|  |  |  | **Group 1** | | | **Group 2** | | | **Group 3** | | | **Group 4** | | |
|  |  |  | **%** | **APP** | **OCC** | **%** | **APP** | **OCC** | **%** | **APP** | **OCC** | **%** | **APP** | **OCC** |
| **Lipid-Lowering CDMP Group (N=5223)** | | | | | | | | | | | | | | |
| 2 | 0 0 | 93343 | 21.9% | 99.3% | 544 | 78.1% | 99.8% | 153 |  |  |  |  |  |  |
| 2 | 0 1 | 93060 | 21.6% | 99.2% | 476 | 78.4% | 99.8% | 147 |  |  |  |  |  |  |
| 2 | 0 2 | 92855 | 21.4% | 99.4% | 561 | 78.6% | 99.8% | 138 |  |  |  |  |  |  |
| 2 | 1 1 | 92832 | 21.6% | 99.6% | 870 | 78.4% | 99.8% | 171 |  |  |  |  |  |  |
| 2 | 1 2 | 92632 | 21.6% | 99.6% | 990 | 78.4% | 99.8% | 143 |  |  |  |  |  |  |
| 2 | 1 0 | 93111 | 21.8% | 99.5% | 749 | 78.2% | 99.8% | 162 |  |  |  |  |  |  |
| 2 | 2 2 | 92633 | 21.6% | 99.5% | 736 | 78.4% | 99.8% | 166 |  |  |  |  |  |  |
| 3 | 0 0 0 | 89179 | 17.7% | 99.7% | 1707 | 16.9% | 94.7% | 86 | 65.4% | 98.3% | 31 |  |  |  |
| 3 | 0 1 1 | 88222 | 17.4% | 100.0% | 11485 | 15.9% | 96.1% | 129 | 66.7% | 98.8% | 43 |  |  |  |
| 3 | 0 1 2 | 88104 | 17.4% | 100.0% | 13353 | 15.7% | 96.1% | 131 | 66.8% | 98.9% | 45 |  |  |  |
| 3 | 0 2 2 | 87884 | 17.4% | 100.0% | 27156 | 15.7% | 96.3% | 135 | 66.8% | 98.8% | 40 |  |  |  |
| 3 | 1 1 1 | 88183 | 17.8% | 99.9% | 5210 | 16.1% | 96.0% | 123 | 66.1% | 98.6% | 37 |  |  |  |
| 3 | 1 1 2 | 88067 | 17.8% | 99.9% | 4970 | 15.9% | 96.2% | 130 | 66.3% | 98.7% | 39 |  |  |  |
| 3 | 1 2 1 | 87959 | 17.8% | 99.8% | 2328 | 16.2% | 95.9% | 117 | 66.0% | 98.6% | 37 |  |  |  |
| 3 | 1 2 2 | 87863 | 17.8% | 99.8% | 2102 | 16.0% | 95.9% | 120 | 66.2% | 98.7% | 38 |  |  |  |
| 3 | 2 1 1 | 88184 | 17.8% | 99.9% | 4235 | 16.1% | 96.0% | 124 | 66.1% | 98.6% | 38 |  |  |  |
| 3 | 2 2 1 | 87961 | 17.8% | 99.9% | 4004 | 16.2% | 95.8% | 117 | 66.0% | 98.6% | 37 |  |  |  |
| 3 | 2 1 2 | 88068 | 17.8% | 99.9% | 4033 | 15.9% | 96.2% | 132 | 66.3% | 98.7% | 39 |  |  |  |
| 4 | 0 2 2 0 | 87519 | 5.2% | 93.3% | 249 | 17.4% | 99.8% | 2604 | 20.6% | 91.0% | 38 | 56.8% | 96.2% | 20 |
| 4 | 0 2 2 1 | 87523 | 5.2% | 93.0% | 240 | 17.4% | 99.8% | 2608 | 20.4% | 91.1% | 38 | 57.0% | 96.3% | 20 |
| 4 | 0 2 2 2 | 87464 | 4.8% | 93.1% | 263 | 17.4% | 99.8% | 2662 | 19.4% | 91.2% | 42 | 58.5% | 96.6% | 21 |
| 4 | 0 2 2 3 | 87266 | 4.5% | 93.0% | 273 | 17.4% | 99.8% | 2729 | 18.6% | 91.1% | 43 | 59.5% | 96.9% | 22 |
| 4 | 0 2 3 3 | 87179 | 4.7% | 93.4% | 281 | 17.4% | 99.8% | 2725 | 19.6% | 91.2% | 41 | 58.3% | 96.7% | 22 |
| 4 | 0 3 3 3 | 87174† | 4.7% | 93.4% | 281 | 17.4% | 99.8% | 2725 | 19.6% | 91.2% | 41 | 58.3% | 96.7% | 22 |
| 4 | 2 1 3 3 | 86401 | 17.4% | 100.0% | 46888 | 20.1% | 89.9% | 33 | 7.9% | 95.7% | 257 | 54.5% | 95.3% | 17 |
| 4 | 2 2 3 3 | 86288† | 17.4% | 100.0% | 104283 | 21.0% | 90.4% | 33 | 7.9% | 95.5% | 247 | 53.7% | 95.1% | 17 |
| 4 | 2 3 3 3 | 86161 | 17.4% | 100.0% | 231301 | 7.9% | 95.4% | 244 | 22.3% | 90.3% | 31 | 52.4% | 94.9% | 17 |
| 4 | 3 3 3 3 | 86167† | 17.4% | 100.0% | 231301 | 7.9% | 95.4% | 244 | 22.3% | 90.3% | 31 | 52.4% | 94.9% | 17 |

| **No. of groups** | **Trajectory shape*** | **BIC** | **% Group Membership** | | | | | | | | | | | |
| --- | --- | --- | --- | --- | --- | --- | --- | --- | --- | --- | --- | --- | --- | --- |
|  |  |  | **Group 1** | | | **Group 2** | | | **Group 3** | | | **Group 4** | | |
|  |  |  | **%** | **APP** | **OCC** | **%** | **APP** | **OCC** | **%** | **APP** | **OCC** | **%** | **APP** | **OCC** |
| **Lipid-Lowering No CDMP Group (N=6357)** | | | | | | | | | | | | | | |
| 2 | 0 0 | 109542 | 31.6% | 99.5% | 463 | 68.4% | 99.8% | 187 |  |  |  |  |  |  |
| 2 | 0 1 | 109059 | 31.3% | 99.4% | 394 | 68.7% | 99.8% | 241 |  |  |  |  |  |  |
| 2 | 0 2 | 108747 | 31.2% | 99.6% | 489 | 68.8% | 99.8% | 240 |  |  |  |  |  |  |
| 2 | 1 2 | 108533 | 31.2% | 99.6% | 489 | 68.8% | 99.8% | 240 |  |  |  |  |  |  |
| 2 | 1 0 | 109323 | 31.1% | 99.7% | 750 | 68.9% | 99.8% | 224 |  |  |  |  |  |  |
| 2 | 2 2 | 108532 | 31.5% | 99.5% | 436 | 68.5% | 99.8% | 219 |  |  |  |  |  |  |
| 3 | 0 0 0 | 104186 | 31.1% | 99.7% | 718 | 68.9% | 99.8% | 247 |  |  |  |  |  |  |
| 3 | 0 1 1 | 102822 | 28.8% | 99.5% | 459 | 17.7% | 94.9% | 85 | 53.5% | 98.2% | 47 |  |  |  |
| 3 | 0 1 2 | 108308 | 27.9% | 100.0% | 10060 | 15.9% | 96.4% | 140 | 56.2% | 98.9% | 68 |  |  |  |
| 3 | 0 2 2 | 102359 | 31.2% | 98.9% | 207 | 20.1% | 91.4% | 41 | 48.7% | 96.2% | 27 |  |  |  |
| 3 | 1 1 1 | 102760 | 27.9% | 100.0% | 14722 | 15.9% | 96.3% | 138 | 56.1% | 98.8% | 66 |  |  |  |
| 3 | 1 1 2 | 102604 | 28.6% | 99.9% | 2448 | 16.1% | 96.6% | 145 | 55.4% | 98.5% | 53 |  |  |  |
| 3 | 1 2 1 | 102457 | 28.6% | 99.9% | 2062 | 15.7% | 96.2% | 135 | 55.7% | 98.7% | 60 |  |  |  |
| 3 | 1 2 2 | 102327 | 28.6% | 99.8% | 1235 | 16.4% | 96.5% | 136 | 55.0% | 98.4% | 52 |  |  |  |
| 3 | 2 1 1 | 102753 | 28.6% | 99.9% | 3118 | 16.1% | 96.6% | 146 | 55.4% | 98.5% | 52 |  |  |  |
| 3 | 2 2 1 | 102451 | 28.6% | 99.8% | 1455 | 16.4% | 96.5% | 138 | 55.0% | 98.4% | 52 |  |  |  |
| 3 | 2 1 2 | 102597 | 28.6% | 99.9% | 2617 | 15.7% | 96.3% | 136 | 55.7% | 98.7% | 60 |  |  |  |
| 4 | 0 2 2 0 | 102051 | 4.1% | 92.3% | 273 | 27.9% | 99.9% | 2096 | 17.9% | 93.1% | 60 | 50.1% | 97.1% | 34 |
| 4 | 0 2 2 1 | 102036 | 4.0% | 91.2% | 246 | 27.9% | 99.9% | 2127 | 17.4% | 93.4% | 65 | 50.7% | 97.4% | 37 |
| 4 | 0 2 2 2 | 101936 | 3.7% | 90.4% | 240 | 27.9% | 99.9% | 2197 | 17.0% | 93.3% | 66 | 51.4% | 97.7% | 41 |
| 4 | 0 2 2 3 | 101761 | 3.5% | 89.9% | 239 | 27.9% | 99.9% | 2267 | 16.7% | 92.5% | 61 | 51.9% | 98.0% | 46 |
| 4 | 0 2 3 3 | 101658 | 3.5% | 90.1% | 247 | 27.9% | 99.9% | 2250 | 17.1% | 93.0% | 64 | 51.4% | 97.9% | 45 |
| 4 | 0 3 3 3 | 101663 | 3.5% | 90.1% | 247 | 27.9% | 99.9% | 2250 | 17.1% | 93.0% | 64 | 51.4% | 97.9% | 45 |
| 4 | 2 1 3 3 | 100577† | 27.9% | 100.0% | 24302 | 7.3% | 96.2% | 317 | 18.2% | 91.2% | 45 | 46.7% | 96.2% | 29 |
| 4 | 2 2 3 3 | 100362 | 27.9% | 100.0% | 138721 | 7.5% | 95.6% | 265 | 18.6% | 90.9% | 43 | 46.0% | 96.1% | 29 |
| 4 | 2 3 3 3 | 100360 | 27.9% | 100.0% | 186555 | 7.7% | 95.7% | 267 | 18.7% | 90.8% | 42 | 45.7% | 96.0% | 29 |
| 4 | 3 3 3 3 | 100365† | 27.9% | 100.0% | 186555 | 7.7% | 95.7% | 267 | 18.7% | 90.8% | 42 | 45.7% | 96.0% | 29 |

| **No. of groups** | **Trajectory shape*** | **BIC** | **% Group Membership** | | | | | | | | | | | |
| --- | --- | --- | --- | --- | --- | --- | --- | --- | --- | --- | --- | --- | --- | --- |
|  |  |  | **Group 1** | | | **Group 2** | | | **Group 3** | | | **Group 4** | | |
|  |  |  | **%** | **APP** | **OCC** | **%** | **APP** | **OCC** | **%** | **APP** | **OCC** | **%** | **APP** | **OCC** |
| **Antihypertensive CDMP Group (N=5223)** | | | | | | | | | | | | | | |
| 2 | 0 1 | 77735 | 24.6% | 100% | 1142 | 75.4% | 100% | 266 |  |  |  |  |  |  |
| 2 | 0 2 | 77583 | 24.5% | 100% | 1556 | 75.5% | 100% | 260 |  |  |  |  |  |  |
| 2 | 1 1 | 77482 | 24.8% | 100% | 1262 | 75.2% | 100% | 396 |  |  |  |  |  |  |
| 2 | 1 2 | 77334 | 24.7% | 100% | 1008 | 75.3% | 100% | 431 |  |  |  |  |  |  |
| 2 | 1 0 | 77738 | 24.9% | 100% | 1430 | 75.1% | 100% | 486 |  |  |  |  |  |  |
| 2 | 2 2 | 77339 | 24.7% | 100% | 1011 | 75.3% | 100% | 427 |  |  |  |  |  |  |
| 3 | 0 0 0 | 73722 | 23.0% | 100% | 1079 | 17.4% | 95% | 92 | 59.6% | 98% | 31 |  |  |  |
| 3 | 0 1 1 | 72996 | 22.8% | 100% | 2350 | 16.3% | 96% | 121 | 61.0% | 98% | 38 |  |  |  |
| 3 | 0 1 2 | 72948 | 22.8% | 100% | 2251 | 16.1% | 96% | 126 | 61.1% | 98% | 39 |  |  |  |
| 3 | 0 2 2 | 72744 | 22.7% | 100% | 1632 | 16.0% | 96% | 127 | 61.3% | 98% | 38 |  |  |  |
| 3 | 1 1 1 | 72904 | 22.8% | 100% | 5293 | 16.3% | 96% | 120 | 60.8% | 98% | 38 |  |  |  |
| 3 | 1 1 2 | 72856 | 22.8% | 100% | 5188 | 16.2% | 96% | 126 | 61.0% | 98% | 39 |  |  |  |
| 3 | 1 2 1 | 72691 | 22.8% | 100% | 4117 | 16.4% | 96% | 116 | 60.8% | 98% | 39 |  |  |  |
| 3 | 1 2 2 | 72657 | 22.8% | 100% | 3892 | 16.2% | 96% | 122 | 61.0% | 98% | 38 |  |  |  |
| 3 | 2 1 1 | 72900 | 22.8% | 100% | 9612 | 16.3% | 96% | 121 | 60.8% | 98% | 38 |  |  |  |
| 3 | 2 2 1 | 72688 | 22.8% | 100% | 5453 | 16.4% | 96% | 116 | 60.8% | 98% | 39 |  |  |  |
| 3 | 2 1 2 | 72852 | 22.8% | 100% | 9822 | 16.2% | 96% | 127 | 61.0% | 98% | 38 |  |  |  |
| 4 | 0 2 2 0 | 72377 | 6.6% | 92% | 153 | 22.7% | 100% | 1855 | 23.2% | 90% | 30 | 47.5% | 95% | 21 |
| 4 | 0 2 2 1 | 72385 | 3.6% | 94% | 404 | 22.3% | 100% | 3227 | 21.1% | 91% | 39 | 53.1% | 96% | 25 |
| 4 | 0 2 2 2 | 72365 | 3.4% | 94% | 401 | 22.3% | 100% | 3246 | 20.5% | 92% | 41 | 53.8% | 97% | 26 |
| 4 | 0 2 2 3 | 72221 | 3.1% | 94% | 455 | 22.3% | 100% | 3298 | 18.9% | 92% | 51 | 55.7% | 97% | 25 |
| 4 | 0 2 3 3 | 72728† | 11.4% | 95% | 137 | 22.8% | 100% | 3106 | 43.0% | 97% | 36 | 22.7% | 91% | 37 |
| 4 | 0 3 3 3 | 72148† | 3.4% | 94% | 424 | 22.3% | 100% | 3343 | 20.6% | 92% | 42 | 53.7% | 97% | 26 |
| 4 | 2 1 3 3 | 71207 | 22.3% | 100% | 157829 | 3.8% | 97% | 710 | 20.4% | 93% | 48 | 53.5% | 97% | 26 |
| 4 | 2 2 3 3 | 71258 | 22.3% | 100% | 94596 | 19.9% | 92% | 48 | 4.1% | 97% | 662 | 53.7% | 97% | 25 |
| 4 | 2 3 3 3 | 71144 | 22.3% | 100% | 271086 | 21.3% | 92% | 42 | 4.4% | 96% | 550 | 52.0% | 96% | 25 |
| 4 | 3 3 3 3 | 71149 | 22.3% | 100% | 271086 | 4.4% | 96% | 550 | 21.3% | 92% | 42 | 52.0% | 96% | 25 |

| **No. of groups** | **Trajectory shape*** | **BIC** | **% Group Membership** | | | | | | | | | | | |
| --- | --- | --- | --- | --- | --- | --- | --- | --- | --- | --- | --- | --- | --- | --- |
|  |  |  | **Group 1** | | | **Group 2** | | | **Group 3** | | | **Group 4** | | |
|  |  |  | **%** | **APP** | **OCC** | **%** | **APP** | **OCC** | **%** | **APP** | **OCC** | **%** | **APP** | **OCC** |
| **Antihypertensive No CDMP Group (N=6357)** | | | | | | | | | | | | | | |
| 2 | 0 0 | 90600 | 35.4% | 100% | 722 | 64.6% | 100% | 267 |  |  |  |  |  |  |
| 2 | 0 1 | 90222 | 35.1% | 100% | 888 | 64.9% | 100% | 248 |  |  |  |  |  |  |
| 2 | 0 2 | 89993 | 35.1% | 100% | 688 | 64.9% | 100% | 302 |  |  |  |  |  |  |
| 2 | 1 2 | 89778 | 35.2% | 100% | 778 | 64.8% | 100% | 282 |  |  |  |  |  |  |
| 2 | 1 0 | 90370 | 35.4% | 100% | 808 | 64.6% | 100% | 310 |  |  |  |  |  |  |
| 3 | 2 2 | 89782 | 35.3% | 100% | 753 | 64.7% | 100% | 307 |  |  |  |  |  |  |
| 3 | 0 0 0 | 85143 | 33.5% | 100% | 996 | 16.1% | 96% | 110 | 50.3% | 98% | 51 |  |  |  |
| 3 | 0 1 1 | 84155 | 33.2% | 100% | 2072 | 15.1% | 96% | 141 | 51.7% | 99% | 65 |  |  |  |
| 3 | 0 1 2 | 84073 | 33.1% | 100% | 2940 | 15.0% | 96% | 133 | 51.8% | 99% | 72 |  |  |  |
| 3 | 0 2 2 | 83793 | 32.9% | 100% | 59355 | 14.9% | 96% | 140 | 52.1% | 99% | 69 |  |  |  |
| 3 | 1 1 1 | 84031 | 33.4% | 100% | 4211 | 15.3% | 96% | 144 | 51.3% | 98% | 62 |  |  |  |
| 3 | 1 1 2 | 83591 | 33.4% | 100% | 3734 | 15.1% | 96% | 135 | 51.4% | 99% | 70 |  |  |  |
| 3 | 1 2 1 | 83756 | 33.4% | 100% | 5416 | 15.5% | 96% | 133 | 51.1% | 98% | 58 |  |  |  |
| 3 | 1 2 2 | 83693 | 33.4% | 100% | 4769 | 15.3% | 96% | 135 | 51.3% | 98% | 62 |  |  |  |
| 3 | 2 1 1 | 84028 | 33.5% | 100% | 2659 | 15.2% | 96% | 149 | 51.3% | 98% | 61 |  |  |  |
| 3 | 2 2 1 | 83753 | 33.4% | 100% | 7168 | 15.5% | 96% | 134 | 51.1% | 98% | 59 |  |  |  |
| 4 | 2 1 2 | 83949 | 33.5% | 100% | 3152 | 15.1% | 96% | 137 | 51.4% | 99% | 69 |  |  |  |
| 4 | 0 2 2 0 | 83445 | 3.7% | 93% | 330 | 32.9% | 100% | 3332 | 17.0% | 92% | 59 | 46.4% | 97% | 40 |
| 4 | 0 2 2 1 | 83445 | 3.6% | 92.7% | 329 | 32.9% | 99.9% | 3346 | 16.7% | 92.5% | 61 | 46.8% | 97.2% | 40 |
| 4 | 0 2 2 2 | 82583 | 17.7% | 89.3% | 36 | 33.1% | 99.9% | 3138 | 9.5% | 95.5% | 203 | 39.7% | 93.6% | 23 |
| 4 | 0 2 2 3 | 82496 | 16.4% | 89.2% | 40 | 32.9% | 100.0% | 23599 | 9.0% | 95.8% | 230 | 41.6% | 94.7% | 26 |
| 4 | 0 2 3 3 | 83202 | 3.3% | 91.5% | 316 | 32.9% | 99.9% | 3463 | 16.2% | 93.4% | 72 | 47.6% | 97.5% | 43 |
| 4 | 0 3 3 3 | 83207 | 3.3% | 91.5% | 316 | 32.9% | 99.9% | 3463 | 16.2% | 93.4% | 72 | 47.6% | 97.5% | 43 |
| 4 | 2 1 3 3 | 82091† | 32.9% | 100.0% | 6029 | 4.6% | 96.3% | 529 | 16.6% | 93.1% | 67 | 45.9% | 97.2% | 41 |
| 4 | 2 2 3 3 | 82004 | 32.9% | 100.0% | 4677 | 5.1% | 95.5% | 392 | 16.8% | 92.8% | 62 | 45.1% | 97.0% | 40 |
| 4 | 2 3 3 3 | 82006† | 32.9% | 100.0% | 4149 | 5.2% | 95.6% | 392 | 16.8% | 92.7% | 62 | 45.1% | 97.0% | 40 |
| 4 | 3 3 3 3 | 82012 | 32.9% | 100.0% | 4146 | 5.2% | 95.6% | 392 | 16.8% | 92.7% | 62 | 45.1% | 97.0% | 40 |

| **No. of groups** | **Trajectory shape*** | **BIC** | **% Group Membership** | | | | | | | | | | | |
| --- | --- | --- | --- | --- | --- | --- | --- | --- | --- | --- | --- | --- | --- | --- |
|  |  |  | **Group 1** | | | **Group 2** | | | **Group 3** | | | **Group 4** | | |
|  |  |  | **%** | **APP** | **OCC** | **%** | **APP** | **OCC** | **%** | **APP** | **OCC** | **%** | **APP** | **OCC** |
| **Antithrombotic CDMP Group (N=4825)** | | | | | | | | | | | | | | |
| 2 | 0 2 | 56972 | 32.0% | 99.9% | 1894 | 68.0% | 99.9% | 577 |  |  |  |  |  |  |
| 2 | 1 1 | 56920 | 32.1% | 99.8% | 1088 | 67.9% | 99.9% | 671 |  |  |  |  |  |  |
| 2 | 1 2 | 54709 | 32.1% | 99.8% | 1047 | 67.9% | 99.9% | 709 |  |  |  |  |  |  |
| 2 | 1 0 | 57010 | 32.1% | 99.9% | 2013 | 67.9% | 99.9% | 556 |  |  |  |  |  |  |
| 2 | 2 2 | 56871 | 32.1% | 99.8% | 1309 | 67.9% | 99.9% | 559 |  |  |  |  |  |  |
| 3 | 0 0 0 | 53078 | 30.9% | 99.9% | 1892 | 13.2% | 95.0% | 123 | 55.9% | 98.5% | 54 |  |  |  |
| 3 | 0 1 1 | 52701 | 30.5% | 99.9% | 3233 | 12.3% | 96.5% | 195 | 57.2% | 98.9% | 66 |  |  |  |
| 3 | 0 1 2 | 52678 | 30.5% | 99.9% | 2704 | 12.2% | 96.4% | 191 | 57.2% | 99.0% | 73 |  |  |  |
| 3 | 0 2 2 | 52583 | 30.5% | 99.9% | 3640 | 12.2% | 96.5% | 197 | 57.2% | 98.9% | 68 |  |  |  |
| 3 | 1 1 1 | 52653 | 30.8% | 99.9% | 2192 | 12.3% | 96.9% | 213 | 56.9% | 98.6% | 56 |  |  |  |
| 3 | 1 1 2 | 52631 | 30.7% | 99.9% | 2630 | 12.2% | 96.7% | 205 | 57.0% | 98.8% | 61 |  |  |  |
| 3 | 1 2 1 | 52561 | 30.7% | 99.9% | 3279 | 12.5% | 96.5% | 190 | 56.8% | 98.6% | 55 |  |  |  |
| 3 | 1 2 2 | 52544 | 30.7% | 99.9% | 2555 | 12.3% | 96.7% | 200 | 57.0% | 98.7% | 59 |  |  |  |
| 3 | 2 1 1 | 52649 | 30.8% | 99.9% | 2571 | 12.3% | 97.0% | 219 | 56.9% | 98.6% | 54 |  |  |  |
| 3 | 2 2 1 | 52558 | 30.7% | 99.9% | 3152 | 12.6% | 96.5% | 187 | 56.7% | 98.6% | 54 |  |  |  |
| 3 | 2 1 2 | 52627 | 30.8% | 99.9% | 3577 | 12.3% | 96.7% | 206 | 57.0% | 98.7% | 60 |  |  |  |
| 4 | 0 2 2 0 | 52068 † | 8.8% | 95.8% | 238 | 30.7% | 99.9% | 2121 | 30.4% | 93.5% | 36 | 30.1% | 98.5% | 142 |
| 4 | 0 2 2 1 | 51822 † | 7.0% | 96.8% | 398 | 30.3% | 100.0% | 6683 | 21.6% | 92.0% | 40 | 41.1% | 94.4% | 25 |
| 4 | 0 2 2 2 | 51828 | 7.0% | 96.8% | 398 | 30.3% | 100.0% | 6683 | 21.6% | 92.0% | 40 | 41.1% | 94.4% | 25 |
| 4 | 0 2 2 3 | 51358 † | 30.4% | 100.0% | 37631 | 7.7% | 97.5% | 450 | 28.2% | 96.9% | 76 | 33.7% | 94.7% | 37 |
| 4 | 0 2 3 3 | 51343 † | 33.7% | 94.7% | 37 | 8.0% | 97.2% | 394 | 31.3% | 98.6% | 143 | 30.3% | 95.3% | 50 |
| 4 | 0 3 3 3 | 51332 | 30.4% | 100.0% | 52203 | 8.1% | 97.1% | 377 | 31.2% | 98.6% | 143 | 30.3% | 95.3% | 49 |
| 4 | 2 1 3 3 | 51421 † | 30.5% | 100.0% | 5110 | 8.0% | 97.2% | 391 | 31.2% | 98.7% | 153 | 30.3% | 95.3% | 49 |
| 4 | 2 2 3 3 | 51330 † | 30.4% | 99.9% | 3573 | 8.0% | 97.0% | 366 | 30.7% | 98.4% | 128 | 30.9% | 94.8% | 44 |
| 4 | 2 3 3 3 | 51121 | 30.4% | 100.0% | 79344 | 7.3% | 97.3% | 464 | 21.9% | 92.4% | 42 | 40.5% | 94.5% | 26 |
| 4 | 3 3 3 3 | 51126 | 30.4% | 100.0% | 80683 | 7.3% | 97.4% | 467 | 21.9% | 92.4% | 42 | 40.5% | 94.5% | 26 |

| **No. of groups** | **Trajectory shape*** | **BIC** | **% Group Membership** | | | | | | | | | | | |
| --- | --- | --- | --- | --- | --- | --- | --- | --- | --- | --- | --- | --- | --- | --- |
|  |  |  | **Group 1** | | | **Group 2** | | | **Group 3** | | | **Group 4** | | |
|  |  |  | **%** | **APP** | **OCC** | **%** | **APP** | **OCC** | **%** | **APP** | **OCC** | **%** | **APP** | **OCC** |
| **Antithrombotic No CDMP Group (N=5878)** | | | | | | | | | | | | | | |
| 2 | 0 1 | 58976 | 43.8% | 99.9% | 2479 | 56.2% | 99.9% | 667 |  |  |  |  |  |  |
| 2 | 0 2 | 58908 | 43.8% | 99.9% | 2147 | 56.2% | 99.9% | 699 |  |  |  |  |  |  |
| 2 | 1 2 | 58771 | 43.8% | 100.0% | 2576 | 56.2% | 99.9% | 692 |  |  |  |  |  |  |
| 2 | 1 0 | 58980 | 43.8% | 99.9% | 1909 | 56.2% | 99.9% | 958 |  |  |  |  |  |  |
| 2 | 2 2 | 58772 | 43.8% | 99.9% | 1746 | 56.2% | 99.9% | 886 |  |  |  |  |  |  |
| 3 | 0 0 0 | 54890 | 43.1% | 99.9% | 1745 | 12.9% | 95.6% | 143 | 44.0% | 97.9% | 61 |  |  |  |
| 3 | 0 1 1 | 54486 | 42.9% | 99.9% | 2206 | 12.4% | 95.3% | 142 | 44.7% | 98.4% | 79 |  |  |  |
| 3 | 0 1 2 | 54477 | 42.9% | 100.0% | 2992 | 12.0% | 95.3% | 146 | 45.1% | 98.4% | 77 |  |  |  |
| 3 | 0 2 2 | 54352 | 42.8% | 100.0% | 4449 | 12.5% | 95.5% | 147 | 44.7% | 98.4% | 79 |  |  |  |
| 3 | 1 1 1 | 54433 | 43.1% | 99.9% | 2140 | 12.4% | 95.9% | 161 | 44.6% | 98.3% | 71 |  |  |  |
| 3 | 1 1 2 | 54425 | 43.1% | 99.9% | 1808 | 12.3% | 95.4% | 145 | 44.7% | 98.4% | 78 |  |  |  |
| 3 | 1 2 1 | 54305 | 43.0% | 100.0% | 2654 | 12.7% | 95.9% | 157 | 44.2% | 98.2% | 71 |  |  |  |
| 3 | 1 2 2 | 54303 | 43.0% | 99.9% | 2309 | 12.5% | 96.0% | 164 | 44.5% | 98.2% | 71 |  |  |  |
| 3 | 2 1 1 | 54433 | 43.1% | 99.9% | 2556 | 12.4% | 95.9% | 162 | 44.5% | 98.2% | 70 |  |  |  |
| 3 | 2 2 1 | 54306 | 43.1% | 99.9% | 2228 | 12.7% | 96.1% | 166 | 44.2% | 98.2% | 68 |  |  |  |
| 3 | 2 1 2 | 54425 | 43.1% | 99.9% | 2176 | 12.3% | 95.5% | 148 | 44.6% | 98.4% | 76 |  |  |  |
| 4 | 0 2 2 0 | 54373 † | 42.9% | 99.9% | 1282 | 12.7% | 95.7% | 152 | 44.5% | 98.4% | 75 | 0.0% | 0.0% | 0 |
| 4 | 0 2 2 1 | 52861 | 42.8% | 99.9% | 1162 | 19.5% | 92.8% | 52 | 6.3% | 96.8% | 451 | 31.4% | 95.5% | 47 |
| 4 | 0 2 2 2 | 52864 | 42.8% | 99.9% | 1162 | 6.3% | 96.9% | 458 | 19.5% | 92.9% | 53 | 31.4% | 95.6% | 48 |
| 4 | 0 2 2 3 | 53073 † | 42.8% | 99.9% | 1161 | 7.0% | 97.0% | 429 | 24.6% | 98.9% | 266 | 25.6% | 95.5% | 66 |
| 4 | 0 2 3 3 | 52732 | 42.8% | 99.9% | 1166 | 6.1% | 97.2% | 515 | 19.4% | 93.3% | 57 | 31.7% | 95.5% | 47 |
| 4 | 0 3 3 3 | 52851 † | 42.8% | 99.9% | 1170 | 6.8% | 97.1% | 447 | 23.6% | 98.5% | 203 | 26.8% | 95.8% | 66 |
| 4 | 2 1 3 3 | 52812 | 42.8% | 99.9% | 1150 | 6.1% | 97.4% | 571 | 19.3% | 93.6% | 59 | 31.9% | 95.5% | 46 |
| 4 | 2 2 3 3 | 52740 | 42.8% | 99.9% | 1168 | 6.1% | 97.2% | 515 | 19.4% | 93.3% | 57 | 31.7% | 95.5% | 47 |
| 4 | 2 3 3 3 | 52725 | 42.8% | 99.9% | 1172 | 6.2% | 97.2% | 506 | 19.5% | 93.3% | 56 | 31.5% | 95.5% | 47 |
| 4 | 3 3 3 3 | 52738 † | 42.8% | 99.9% | 1172 | 6.3% | 97.1% | 489 | 19.5% | 93.3% | 57 | 31.5% | 95.5% | 47 |

APP: Average Posterior Probability; BIC: Bayesian Information Criterion; OCC: Odds of Correct Classification.

* 0 = Zero order; 1 = Linear; 2 = Quadratic; 3 = Cubic.

† Model convergence error.

Highlighted rows indicate the final selected models.

Percentages (%) represent the proportion of patients assigned to each trajectory.

**Table S5. Median proportion of days covered, by medication adherence trajectory and chronic disease management claim**

|  | **Trajectory 1 Non-Use** | | **Trajectory 2 Declining Adherence** | | **Trajectory 3 High Adherence** | | **Trajectory 4 Near-Perfect Adherence** | |
| --- | --- | --- | --- | --- | --- | --- | --- | --- |
|  | n (%) | Median PDC (IQR) | n (%) | Median PDC  (IQR) | n (%) | Median PDC  (IQR) | n (%) | Median PDC  (IQR) |
| Antihypertensive, N=11 580 | | | | | | | | |
| Overall | 3257 (28.1) | 0 (0–0) | 557 (4.8) | 24.7 (15.3–35.1) | 2184 (18.9) | 74.0 (56.8–83.3) | 5582 (48.2) | 93.4 (90.1–96.4) |
| No CDM claim | 2094 (32.9) | 0 (0–0) | 327 (5.1) | 24.7 (15.3–33.7) | 1069 (16.8) | 72.1 (54.0–82.2) | 2867 (45.1) | 93.4 (89.9–96.2) |
| CDM claim | 1163 (22.3)* | 0 (0–0) | 230 (4.4) | 24.7 (15.3–38.1) | 1115 (21.4)* | 74.5 (57.5–84.4) | 2715 (52.0)* | 93.7 (90.4–96.4) |
| Antithrombotic, N=10 703† | | | | | | | | |
| Overall | 3979 (37.2) | 0 (0–0) | 711(6.6) | 41.1 (23.0–49.9) | 2198 (20.5) | 86.6 (78.4–91.2) | 3815 (35.6) | 94.2 (90.4–97.0) |
| No CDM claim | 2514 (42.8) | 0 (0–0) | 360 (6.1) | 40.7 (23.0–49.3) | 1143 (19.5) | 86.6 (77.0–91.5) | 1861 (31.7) | 94.2 (90.4–97.3) |
| CDM claim | 1465 (30.4)* | 0 (0–0) | 351 (7.3)* | 41.9 (20.0–50.4) | 1055 (21.9)* | 86.3 (79.5–91.2) | 1954 (40.5)* | 94.2 (90.1–97.0) |
| Lipid-Lowering, N=11 580 | | | | | | | | |
| Overall | 2684 (23.2) | 0 (0–0) | 901 (7.8) | 24.7 (16.4–40.8) | 2350 (20.3) | 74.0 (58.9–82.2) | 5645 (48.8) | 92.3 (88.5–95.3) |
| No CDM claim | 1775 (27.9) | 0 (0–0) | 489 (7.7) | 24.7 (8.2–35.3) | 1187 (18.7) | 71.8 (57.5–80.5) | 2906 (45.7) | 92.1 (87.7–95.1) |
| CDM claim | 909 (17.4)* | 0 (0–0) | 412 (7.9) | 24.7 (16.4–41.1) | 1163 (22.3)* | 75.9 (64.9–83.6) | 2739 (52.4)* | 92.6 (89.3–95.6) |

CDM denotes Chronic Disease Management; IQR, interquartile range; PDC, proportion of days covered.

**p* <0.05 for comparison of trajectory membership, by whether or not they had a chronic disease management Medicare claim.

† Excludes participants with intracerebral haemorrhage.

**Table S6**. **Participant characteristics, by medication and adherence trajectory**

|  | **Antihypertensive Medication Trajectories** | | | | **Antithrombotic Medication Trajectories** | | | | **Lipid-Lowering Medication Trajectories** | | | |
| --- | --- | --- | --- | --- | --- | --- | --- | --- | --- | --- | --- | --- |
|  | Non-Use | Declining | High | Near-Perfect | Non-Use | Declining | High | Near-Perfect | Non-Use | Declining | High | Near-Perfect |
|  | N= 3257 | N= 557 | N= 2184 | N= 5582 | N= 3979 | N= 711 | N= 2198 | N= 3815 | N= 2684 | N= 901 | N= 2350 | N= 5645 |
|  | n (%) | n (%) | n (%) | n (%) | n (%) | n (%) | n (%) | n (%) | n (%) | n (%) | n (%) | n (%) |
| **At admission** |  |  |  |  |  |  |  |  |  |  |  |  |
| Median age in years (Q1–Q3) | 65.3  (52.1–76.8) | 69.4  (57.2–79.0) | 68.8  (59.1–77.5) | 72.6  (64.0–79.8) | 65.1  (53.9–75.4) | 68.1  (58.1–78.1) | 72.0  (63.2–79.3) | 73.8  (64.9–80.8) | 66.1  (52.1–78.4) | 66.3  (55.6–76.4) | 68.4  (59.0–77.4) | 72.5  (63.8–79.5) |
| Female | 1462 (44.9) | 215 (38.6) | 820 (37.5) | 2377 (42.6) | 1786 (44.9) | 242 (34.0) | 827 (37.6) | 1643 (43.1) | 1307 (48.7) | 362 (40.2) | 883 (37.6) | 2322 (41.1) |
| Married | 2149 (66.0) | 344 (61.8) | 1465 (67.1) | 3731 (66.8) | 2594 (65.2) | 449 (63.2) | 1484 (67.5) | 2544 (66.7) | 1681 (62.6) | 591 (65.6) | 1564 (66.6) | 3853 (68.3) |
| Metropolitan residence | 2165 (66.5) | 366 (65.7) | 1506 (69.0) | 3758 (67.3) | 2677 (67.3) | 465 (65.4) | 1454 (66.2) | 2603 (68.2) | 1745 (65.0) | 627 (69.6) | 1599 (68.0) | 3824 (67.7) |
| Private health insurance | 1276 (39.2) | 192 (34.5) | 789 (36.1) | 2251 (40.3) | 1428 (35.9) | 234 (32.9) | 923 (42.0) | 1555 (40.8) | 1062 (39.6) | 297 (33.0) | 817 (34.8) | 2332 (41.3) |
| Received concessional medication benefits | 1888 (58.0) | 385 (69.1) | 1419 (65.0) | 4214 (75.5) | 2267 (57.0) | 493 (69.3) | 1599 (72.7) | 2987 (78.3) | 1511 (56.3) | 557 (61.8) | 1549 (65.9) | 4289 (76.0) |
| Type of stroke |  |  |  |  |  |  |  |  |  |  |  |  |
| Intracerebral haemorrhage | 217 (6.7) | 46 (8.3) | 157 (7.2) | 457 (8.2) | - | - | - | - | 373 (13.9) | 37 (4.1) | 130 (5.5) | 337 (6.0) |
| Ischaemic | 1902 (58.4) | 350 (62.8) | 1400 (64.1) | 3589 (64.3) | 2414 (60.7) | 463 (65.1) | 1608 (73.2) | 2756 (72.2) | 1392 (51.9) | 558 (61.9) | 1523 (64.8) | 3768 (66.7) |
| TIA | 1036 (31.8) | 146 (26.2) | 573 (26.2) | 1396 (25.0) | 1427 (35.9) | 219 (30.8) | 542 (24.7) | 963 (25.2) | 826 (30.8) | 283 (31.4) | 637 (27.1) | 1405 (24.9) |
| Undetermined | 102 (3.1) | 15 (2.7) | 54 (2.5) | 140 (2.5) | 138 (3.5) | 29 (4.1) | 48 (2.2) | 96 (2.5) | 93 (3.5) | 23 (2.6) | 60 (2.6) | 135 (2.4) |
| Previous stroke | 509 (15.6) | 112 (20.1) | 429 (19.6) | 1070 (19.2) | 489 (12.3) | 156 (21.9) | 464 (21.1) | 859 (22.5) | 429 (16.0) | 173 (19.2) | 468 (19.9) | 1050 (18.6) |
| Unable to walk on admission | 1419 (43.6) | 239 (42.9) | 952 (43.6) | 2582 (46.3) | 1515 (38.1) | 293 (41.2) | 995 (45.3) | 1803 (47.3) | 1156 (43.1) | 367 (40.7) | 1026 (43.7) | 2643 (46.8) |
| Interpreter needed | 85 (2.6) | 26 (4.7) | 74 (3.4) | 219 (3.9) | 137 (3.4) | 18 (2.5) | 73 (3.3) | 131 (3.4) | 64 (2.4) | 31 (3.4) | 97 (4.1) | 212 (3.8) |
| Treated in a stroke unit | 2557 (78.5) | 458 (82.2) | 1799 (82.4) | 4656 (83.4) | 3077 (77.3) | 560 (78.8) | 1859 (84.6) | 3218 (84.4) | 2087 (77.8) | 725 (80.5) | 1939 (82.5) | 4719 (83.6) |
| Received in-patient rehabilitation | 833 (25.6) | 149 (26.8) | 619 (28.3) | 1818 (32.6) | 851 (21.4) | 185 (26.0) | 637 (29.0) | 1288 (33.8) | 698 (26.0) | 228 (25.3) | 639 (27.2) | 1854 (32.8) |
| Comorbidities |  |  |  |  |  |  |  |  |  |  |  |  |
| Atrial fibrillation | 643 (19.7) | 118 (21.2) | 407 (18.6) | 1315 (23.6) | 249 (6.3) | 121 (17.0) | 650 (29.6) | 1298 (34.0) | 527 (19.6) | 140 (15.5) | 439 (18.7) | 1377 (24.4) |
| Cancer (excl. skin) | 218 (6.7) | 28 (5.0) | 139 (6.4) | 372 (6.7) | 227 (5.7) | 45 (6.3) | 154 (7.0) | 278 (7.3) | 149 (5.6) | 55 (6.1) | 138 (5.9) | 415 (7.4) |
| Chronic respiratory disease | 211 (6.5) | 44 (7.9) | 140 (6.4) | 288 (5.2) | 199 (5.0) | 54 (7.6) | 138 (6.3) | 233 (6.1) | 145 (5.4) | 53 (5.9) | 145 (6.2) | 340 (6.0) |
| Dementia | 62 (1.9) | 16 (2.9) | 32 (1.5) | 83 (1.5) | 57 (1.4) | 19 (2.7) | 28 (1.3) | 77 (2.0) | 60 (2.2) | 10 (1.1) | 33 (1.4) | 90 (1.6) |
| Diabetes | 476 (14.6) | 138 (24.8) | 625 (28.6) | 1598 (28.6) | 767 (19.3) | 220 (30.9) | 585 (26.6) | 1085 (28.4) | 366 (13.6) | 255 (28.3) | 669 (28.5) | 1547 (27.4) |
| Congestive heart failure | 182 (5.6) | 51 (9.2) | 205 (9.4) | 470 (8.4) | 125 (3.1) | 62 (8.7) | 182 (8.3) | 458 (12.0) | 193 (7.2) | 64 (7.1) | 161 (6.9) | 490 (8.7) |
| Myocardial infarction | 193 (5.9) | 46 (8.3) | 189 (8.7) | 486 (8.7) | 167 (4.2) | 85 (12.0) | 203 (9.2) | 396 (10.4) | 113 (4.2) | 73 (8.1) | 176 (7.5) | 552 (9.8) |
| Dyslipidaemia | 1408 (43.2) | 298 (53.5) | 1300 (59.5) | 3548 (63.6) | 1716 (43.1) | 458 (64.4) | 1417 (64.5) | 2524 (66.2) | 775 (28.9) | 537 (59.6) | 1454 (61.9) | 3788 (67.1) |
| Hypertension | 1312 (40.3) | 375 (67.3) | 1587 (72.7) | 4397 (78.8) | 2069 (52.0) | 495 (69.6) | 1543 (70.2) | 2870 (75.2) | 1434 (53.4) | 580 (64.4) | 1556 (66.2) | 4101 (72.6) |
| Anxiety or depression | 309 (9.5) | 52 (9.3) | 204 (9.3) | 496 (8.9) | 368 (9.2) | 75 (10.5) | 186 (8.5) | 329 (8.6) | 266 (9.9) | 81 (9.0) | 201 (8.6) | 513 (9.1) |
| Liver disease | 91 (2.8) | 11 (2.0) | 53 (2.4) | 77 (1.4) | 75 (1.9) | 18 (2.5) | 35 (1.6) | 66 (1.7) | 86 (3.2) | 22 (2.4) | 39 (1.7) | 85 (1.5) |
| Renal disease | 209 (6.4) | 52 (9.3) | 214 (9.8) | 554 (9.9) | 245 (6.2) | 66 (9.3) | 208 (9.5) | 435 (11.4) | 174 (6.5) | 96 (10.7) | 213 (9.1) | 546 (9.7) |
| Smoking history | 1754 (53.9) | 319 (57.3) | 1261 (57.7) | 3054 (54.7) | 2163 (54.4) | 445 (62.6) | 1239 (56.4) | 2092 (54.8) | 1299 (48.4) | 524 (58.2) | 1380 (58.7) | 3185 (56.4) |
| Previous fracture | 525 (16.1) | 82 (14.7) | 321 (14.7) | 811 (14.5) | 534 (13.4) | 91 (12.8) | 354 (16.1) | 609 (16.0) | 403 (15.0) | 126 (14.0) | 335 (14.3) | 875 (15.5) |
| Previous fall | 367 (11.3) | 59 (10.6) | 227 (10.4) | 577 (10.3) | 354 (8.9) | 75 (10.5) | 211 (9.6) | 446 (11.7) | 287 (10.7) | 84 (9.3) | 260 (11.1) | 599 (10.6) |
| Low blood pressure | 469 (14.4) | 88 (15.8) | 298 (13.6) | 724 (13.0) | 413 (10.4) | 101 (14.2) | 329 (15.0) | 609 (16.0) | 323 (12.0) | 124 (13.8) | 287 (12.2) | 845 (15.0) |
| Mean Charlson Comorbidity Index (SD) | 1.5 (1.8) | 1.8 (1.9) | 1.8 (1.8) | 1.7 (1.7) | 1.4 (1.7) | 1.8 (1.8) | 1.8 (1.8) | 1.9 (1.8) | 1.5 (1.8) | 1.8 (2.0) | 1.6 (1.7) | 1.8 (1.8) |
| Regular primary care visits (≥1 visit/6 months) | 2237 (68.7) | 408 (73.2) | 1629 (74.6) | 4487 (80.4) | 2723 (68.4) | 520 (73.1) | 1730 (78.7) | 3138 (82.3) | 1835 (68.4) | 630 (69.9) | 1715 (73.0) | 4581 (81.2) |
| Continuity with same primary care physician | 666 (20.4) | 105 (18.9) | 488 (22.3) | 1431 (25.6) | 788 (19.8) | 147 (20.7) | 537 (24.4) | 1016 (26.6) | 522 (19.4) | 173 (19.2) | 514 (21.9) | 1481 (26.2) |
| **0–6 months post-admission** |  |  |  |  |  |  |  |  |  |  |  |  |
| Median primary care visits (Q1–Q3) | 9 (6–14) | 10 (6–15) | 10 (7–15) | 11 (7–15) | 9 (6–14) | 10 (6–15) | 11 (7–15) | 11 (7–16) | 9 (5–14) | 10 (7–15) | 10 (7–15) | 11 (7–15) |
| Specialist visit with |  |  |  |  |  |  |  |  |  |  |  |  |
| Cardiologist | 1012 (31.1) | 196 (35.2) | 728 (33.3) | 1929 (34.6) | 1337 (33.6) | 240 (33.8) | 820 (37.3) | 1305 (34.2) | 778 (29.0) | 307 (34.1) | 813 (34.6) | 1967 (34.8) |
| Geriatrician | 193 (5.9) | 35 (6.3) | 128 (5.9) | 385 (6.9) | 159 (4.0) | 42 (5.9) | 168 (7.6) | 296 (7.8) | 188 (7.0) | 41 (4.6) | 135 (5.7) | 377 (6.7) |
| Neurologist | 1034 (31.7) | 154 (27.6) | 710 (32.5) | 1928 (34.5) | 1381 (34.7) | 198 (27.8) | 785 (35.7) | 1154 (30.2) | 846 (31.5) | 262 (29.1) | 781 (33.2) | 1937 (34.3) |
| Rehab Physician | 277 (8.5) | 48 (8.6) | 209 (9.6) | 699 (12.5) | 322 (8.1) | 51 (7.2) | 242 (11.0) | 452 (11.8) | 242 (9.0) | 77 (8.5) | 221 (9.4) | 693 (12.3) |

SD denotes standard deviation; Q1, 25th percentile; Q3, 75th percentile; and TIA, transient ischaemic attack.

*Measured in the 1-year period before the index admission.

**Table S7. Effect of having a claim for chronic disease management on trajectories of antihypertensive adherence, overall and by sub-groups**

|  | **Trajectory 1 –  Non use** | | **Trajectory 2 –  Declining adherence** | | **Trajectory 3 –  High adherence** | | **Trajectory 4 –  Near-perfect adherence** | |
| --- | --- | --- | --- | --- | --- | --- | --- | --- |
|  | **N= 3257** | | **N= 557** | | **N= 2184** | | **N= 5582** | |
|  | aOR (95% CI)* | *P*_int._ | aOR (95% CI)* | *P*_int._ | aOR (95% CI)* | *P*_int._ | aOR (95% CI)* | *P*_int._ |
| **Overall** | 0.83 (0.77 – 0.90) |  | 0.84 (0.65 – 1.08) |  | 1.33 (1.24 – 1.44) |  | 1.00 (0.93 – 1.07) |  |
| **Sub-groups** |  |  |  |  |  |  |  |  |
| Age |  |  |  |  |  |  |  |  |
| <75 years | 0.82 (0.74 – 0.90) | 0.68 | 0.92 (0.73 – 1.15) | 0.16 | 1.30 (1.16 – 1.44) | 0.16 | 1.01 (0.95 – 1.08) | 0.62 |
| 75+ years | 0.85 (0.73 – 0.98) |  | 0.73 (0.49 – 1.09) |  | 1.42 (1.31 – 1.54) |  | 0.98 (0.86 – 1.12) |  |
| Sex |  |  |  |  |  |  |  |  |
| Male | 0.82 (0.73 – 0.91) | 0.76 | 0.86 (0.70 – 1.05) | 0.77 | 1.28 (1.12 – 1.46) | 0.32 | 1.02 (0.93 – 1.13) | 0.61 |
| Female | 0.84 (0.73 – 0.97) |  | 0.82 (0.55 – 1.21) |  | 1.42 (1.27 – 1.59) |  | 0.97 (0.84 – 1.12) |  |
| Type of event |  |  |  |  |  |  |  |  |
| Stroke | 0.93 (0.83 – 1.03) | >0.05 | 0.73 (0.51 – 1.05) | 0.08 | 1.38 (1.19 – 1.61) | 0.65 | 0.93 (0.83 – 1.04) | 0.14 |
| TIA | 0.77 (0.68 – 0.88) |  | 0.91 (0.74 – 1.13) |  | 1.31 (1.14 – 1.49) |  | 1.05 (0.95 – 1.14) |  |
| Newly dispensed antihypertensive medication post-stroke^†^ | | | |  |  |  |  |  |
| No | 0.76 (0.62 – 0.94) | 0.31 | 0.85 (0.61 – 1.20) | 0.87 | 1.31 (1.18 – 1.46) | 0.85 | 0.92 (0.81 – 1.05) | 0.18 |
| Yes | 0.84 (0.78 – 0.90) |  | 0.83 (0.63 – 1.09) |  | 1.35 (1.12 – 1.63) |  | 1.05 (0.96 – 1.16) |  |
| Regularly visited their primary care physician^‡^ | | | | |  |  |  |  |
| No | 0.90 (0.79 – 1.02) | >0.05 | 0.84 (0.62 – 1.13) | 0.96 | 1.29 (1.16 – 1.43) | 0.35 | 0.96 (0.85 – 1.08) | 0.34 |
| Yes | 0.67 (0.55 – 0.83) |  | 0.85 (0.62 – 1.15) |  | 1.47 (1.18 – 1.85) |  | 1.14 (0.88 – 1.48) |  |
| Previous claim for a chronic disease management plan before the exposure period^§^ | | | | | | | | |
| No (i.e., new user) | 0.91 (0.79 – 1.04) | 0.74 | 0.85 (0.65 – 1.11) | 0.38 | 1.52 (1.35 – 1.71) | >0.05 | 0.85 (0.75 – 0.96) | 0.28 |
| Yes | 0.94 (0.83 – 1.05) |  | 0.72 (0.53 – 0.99) |  | 1.26 (1.05 – 1.50) |  | 0.97 (0.85 – 1.12) |  |

aOR denotes adjusted odds ratio; CI, confidence interval; *P*_int_; interaction *P* value; and TIA, transient ischaemic attack.

* Derived using multi-level logistic regression, adjusted using inverse probability treatment weights.

† Based on whether the patient had evidence of the medication being dispensed in the 90-day period before stroke admission.

‡ Based on whether the patient visited their primary care physician at least every 6 months in the 2-year period before stroke admission.

§ Based on the presence of a claim for chronic disease management in the 1-year period before, and 6-month period after, stroke admission.

**Table S8. Effect of having a claim for chronic disease management on trajectories of antithrombotic adherence, overall and by sub-groups**

|  | **Trajectory 1 –  Non use** | | **Trajectory 2 –  Declining adherence** | | **Trajectory 3 –  High adherence** | | **Trajectory 4 –  Near-perfect adherence** | |
| --- | --- | --- | --- | --- | --- | --- | --- | --- |
|  | **N= 3979** | | **N= 711** | | **N= 2198** | | **N= 3815** | |
|  | aOR (95% CI)* | *P*_int._ | aOR (95% CI)* | *P*_int._ | aOR (95% CI)* | *P*_int._ | aOR (95% CI)* | *P*_int._ |
| **Overall** | 0.81 (0.76 – 0.86) |  | 1.17 (0.93 – 1.47) |  | 1.03 (0.96 – 1.11) |  | 1.16 (1.08 – 1.25) |  |
| **Sub-groups** |  |  |  |  |  |  |  |  |
| Age |  |  |  |  |  |  |  |  |
| <75 years | 0.78 (0.70 – 0.87) | 0.36 | 1.17 (0.90 – 1.52) | 0.97 | 1.02 (0.93 – 1.12) | 0.89 | 1.24 (1.09 – 1.41) | 0.22 |
| 75+ years | 0.86 (0.75 – 0.99) |  | 1.16 (0.92 – 1.45) |  | 1.04 (0.87 – 1.24) |  | 1.05 (0.89 – 1.25) |  |
| Sex |  |  |  |  |  |  |  |  |
| Male | 0.77 (0.70 – 0.86) | 0.31 | 1.17 (0.84 – 1.64) | 0.95 | 0.95 (0.85 – 1.05) | 0.02 | 1.27 (1.12 – 1.45) | 0.74 |
| Female | 0.85 (0.76 – 0.94) |  | 1.16 (0.90 – 1.49) |  | 1.18 (1.04 – 1.34) |  | 1.03 (0.88 – 1.19) |  |
| Type of event |  |  |  |  |  |  |  |  |
| Stroke | 0.75 (0.70 – 0.80) | 0.02 | 1.33 (1.04 – 1.70) | 0.52 | 1.07 (0.92 – 1.24) | 0.66 | 1.22 (1.08 – 1.37) | 0.20 |
| TIA | 0.84 (0.77 – 0.93) |  | 1.09 (0.84 – 1.41) |  | 1.01 (0.90 – 1.14) |  | 1.13 (1.04 – 1.24) |  |
| Newly dispensed antithrombotic medication post-stroke* | | | |  |  |  |  |  |
| No | 0.93 (0.70 – 1.23) | 0.36 | 0.99 (0.67 – 1.44) | 0.13 | 1.00 (0.78 – 1.29) | 0.85 | 1.04 (0.81 – 1.33) | 0.36 |
| Yes | 0.79 (0.74 – 0.85) |  | 1.23 (0.94 – 1.60) |  | 1.03 (0.95 – 1.12) |  | 1.20 (1.08 – 1.34) |  |
| Regularly visited their primary care physician^‡^ | | |  |  |  |  |  |  |
| No | 0.86 (0.79 – 0.93) | 0.08 | 1.16 (0.97 – 1.38) | 0.81 | 1.02 (0.94 – 1.10) | 0.59 | 1.10 (1.01 – 1.20) | 0.044 |
| Yes | 0.70 (0.58 – 0.84) |  | 1.20 (0.80 – 1.80) |  | 1.05 (0.94 – 1.19) |  | 1.40 (1.14 – 1.73) |  |
| Previous claim for a chronic disease management plan before the exposure period^§^ | | | | | | | | |
| No (i.e., new user) | 0.92 (0.77 – 1.11) | 0.75 | 1.29 (0.95 – 1.76) | 0.44 | 0.91 (0.83 – 1.00) | 0.09 | 1.08 (0.90 – 1.29) | 0.98 |
| Yes | 0.89 (0.78 – 1.01) |  | 1.11 (0.82 – 1.50) |  | 1.01 (0.92 – 1.10) |  | 1.08 (0.91 – 1.28) |  |

aOR denotes adjusted odds ratio; CI, confidence interval; *P*_int_; interaction *P* value; and TIA, transient ischaemic attack.

* Derived using multi-level logistic regression, adjusted using inverse probability treatment weights.

† Based on whether the patient had evidence of the medication being dispensed in the 90-day period before stroke admission.

‡ Based on whether the patient visited their primary care physician at least every 6 months in the 2-year period before stroke admission.

§ Based on the presence of a claim for chronic disease management in the 1-year period before, and 6-month period after, stroke admission.

**Table S9. Effect of having a claim for chronic disease management on trajectories of lipid-lowering adherence, overall and by sub-groups**

|  | **Trajectory 1 –  Non use** | | **Trajectory 2 –  Declining adherence** | | **Trajectory 3 –  High adherence** | | **Trajectory 4 –  Near-perfect adherence** | |
| --- | --- | --- | --- | --- | --- | --- | --- | --- |
|  | **N= 2684** | | **N= 901** | | **N= 2530** | | **N= 5645** | |
|  | aOR (95% CI)* | *P*_int._ | aOR (95% CI)* | *P*_int._ | aOR (95% CI)* | *P*_int._ | aOR (95% CI)* | *P*_int._ |
| **Overall** | 0.77 (0.70 – 0.84) |  | 1.07 (0.92 – 1.25) |  | 1.26 (1.16 – 1.37) |  | 1.01 (0.94 – 1.08) |  |
| **Sub-groups** |  |  |  |  |  |  |  |  |
| Age |  |  |  |  |  |  |  |  |
| <75 years | 0.71 (0.63 – 0.79) | 0.041 | 1.12 (0.90 – 1.39) | 0.38 | 1.30 (1.19 – 1.43) | 0.30 | 1.03 (0.94 – 1.12) | 0.52 |
| 75+ years | 0.90 (0.75 – 1.08) |  | 0.97 (0.78 – 1.20) |  | 1.18 (1.01 – 1.37) |  | 0.98 (0.88 – 1.11) |  |
| Sex |  |  |  |  |  |  |  |  |
| Male | 0.78 (0.70 – 0.87) | 0.43 | 1.08 (0.86 – 1.36) | 0.89 | 1.24 (1.13 – 1.36) | 0.52 | 0.99 (0.90 – 1.09) | 0.32 |
| Female | 0.75 (0.68 – 0.82) |  | 1.06 (0.83 – 1.37) |  | 1.30 (1.14 – 1.49) |  | 1.04 (0.96 – 1.14) |  |
| Type of event |  |  |  |  |  |  |  |  |
| Stroke | 0.80 (0.70 – 0.92) | 0.43 | 1.01 (0.86 – 1.20) | 0.46 | 1.21 (1.09 – 1.34) | 0.25 | 1.07 (0.96 – 1.18) | 0.19 |
| TIA | 0.74 (0.66 – 0.82) |  | 1.11 (0.90 – 1.38) |  | 1.29 (1.18 – 1.41) |  | 0.98 (0.89 – 1.07) |  |
| Newly dispensed lipid-lowering medication post-stroke* | | | |  |  |  |  |  |
| No | 0.75 (0.61 – 0.91) | 0.83 | 0.89 (0.77 – 1.02) | 0.02 | 1.27 (1.13 – 1.43) | 0.74 | 0.93 (0.86 – 1.00) | 0.48 |
| Yes | 0.77 (0.69 – 0.85) |  | 1.20 (0.96 – 1.50) |  | 1.24 (1.12 – 1.38) |  | 1.04 (0.93 – 1.16) |  |
| Regularly visited their primary care physician^‡^ | | |  |  |  |  |  |  |
| No | 0.78 (0.69 – 0.87) | 0.66 | 1.08 (0.90 – 1.30) | 0.94 | 1.22 (1.12 – 1.34) | 0.35 | 1.01 (0.90 – 1.15) | 0.89 |
| Yes | 0.74 (0.64 – 0.87) |  | 1.06 (0.70 – 1.61) |  | 1.37 (1.11 – 1.70) |  | 0.99 (0.75 – 1.30) |  |
| Previous claim for a chronic disease management plan before the exposure period^§^ | | | | | | | | |
| No (i.e., new user) | 0.98 (0.89 – 1.08) | 0.02 | 1.14 (0.87 – 1.48) | 0.41 | 1.17 (1.05 – 1.32) | 0.06 | 0.88 (0.81 – 0.97) | 0.13 |
| Yes | 0.83 (0.74 – 0.93) |  | 0.99 (0.79 – 1.24) |  | 1.25 (1.12 – 1.40) |  | 0.97 (0.90 – 1.05) |  |

aOR denotes adjusted odds ratio; CI, confidence interval; *P*_int_; interaction *P* value; and TIA, transient ischaemic attack.

* Derived using multi-level logistic regression, adjusted using inverse probability treatment weights.

† Based on whether the patient had evidence of the medication being dispensed in the 90-day period before stroke admission.

‡ Based on whether the patient visited their primary care physician at least every 6 months in the 2-year period before stroke admission.

§ Based on the presence of a claim for chronic disease management in the 1-year period before, and 6-month period after, stroke admission.

**Table S10. Association of having a chronic disease management claim on trajectories of medication adherence, by medication group and timing of chronic disease management claim during the exposure period***

|  | **Trajectory 1 –  Non use** | | **Trajectory 2 –  Declining adherence** | | **Trajectory 3 –  High adherence** | | **Trajectory 4 –  Near-perfect adherence** | |
| --- | --- | --- | --- | --- | --- | --- | --- | --- |
|  | n/N | aOR (95% CI)^†^ | n/N | aOR (95% CI)^†^ | n/N | aOR (95% CI)^†^ | n/N | aOR (95% CI)^†^ |
| **Antihypertensive medications** | | | | | | | | |
| No CDM claim | 2094/6357 | Ref | 327/6357 | Ref | 1069/6357 | Ref | 2867/6357 | Ref |
| CDM claim during first quarter | 469/2012 | 0.87 (0.77 – 0.98) | 86/2012 | 0.84 (0.60 – 1.17) | 391/2012 | 1.22 (1.08 – 1.39) | 1066/2012 | 1.02 (0.94 – 1.12) |
| CDM claim during second quarter | 341/1524 | 0.82 (0.72 – 0.95) | 71/1524 | 0.94 (0.78 – 1.15) | 337/1524 | 1.36 (1.15 – 1.60) | 775/1524 | 0.98 (0.87 – 1.10) |
| CDM claim during third quarter | 199/941 | 0.83 (0.70 – 0.97) | 43/941 | 0.79 (0.55 – 1.15) | 226/941 | 1.52 (1.35 – 1.71) | 473/941 | 0.93 (0.85 – 1.02) |
| CDM claim during fourth quarter | 154/746 | 0.75 (0.63 – 0.90) | 30/746 | 0.71 (0.44 – 1.16) | 161/746 | 1.35 (1.17 – 1.57) | 401/746 | 1.10 (0.98 – 1.22) |
| **Antithrombotic medications** | | | | | | | | |
| No CDM claim | 2514/5878 | Ref | 360/5878 | Ref | 1143/5878 | Ref | 1861/5878 | Ref |
| CDM claim during first quarter | 551/1875 | 0.77 (0.69 – 0.86) | 137/1875 | 1.17 (0.87 – 1.56) | 430/1875 | 1.10 (0.96 – 1.25) | 757/1875 | 1.16 (1.04 – 1.30) |
| CDM claim during second quarter | 448/1425 | 0.84 (0.75 – 0.94) | 103/1425 | 1.14 (0.80 – 1.64) | 297/1425 | 0.97 (0.85 – 1.11) | 577/1425 | 1.17 (1.04 – 1.33) |
| CDM claim during third quarter | 260/856 | 0.83 (0.74 – 0.93) | 60/856 | 1.16 (0.93 – 1.44) | 183/856 | 0.96 (0.81 – 1.15) | 353/856 | 1.18 (1.03 – 1.36) |
| CDM claim during fourth quarter | 206/669 | 0.81 (0.69 – 0.95) | 51/669 | 1.22 (0.92 – 1.62) | 145/669 | 1.06 (0.85 – 1.32) | 267/669 | 1.12 (1.01 – 1.24) |
| **Lipid-lowering medications** | | | | | | | | |
| No CDM claim | 1775/6357 | Ref | 489/6357 | Ref | 1187/6357 | Ref | 2906/6357 | Ref |
| CDM claim during first quarter | 332/2012 | 0.71 (0.59 – 0.84) | 150/2012 | 1.00 (0.83 – 1.21) | 453/2012 | 1.34 (1.16 – 1.55)^‡^ | 1077/2012 | 1.04 (0.92 – 1.17) |
| CDM claim during second quarter | 260/1524 | 0.75 (0.68 – 0.83) | 116/1524 | 1.07 (0.82 – 1.38) | 348/1524 | 1.25 (1.13 – 1.38)^‡^ | 800/1524 | 1.04 (0.97 – 1.10) |
| CDM claim during third quarter | 169/941 | 0.82 (0.70 – 0.95) | 78/941 | 1.06 (0.80 – 1.41) | 222/941 | 1.37 (1.23 – 1.54)^‡^ | 472/941 | 0.92 (0.82 – 1.03) |
| CDM claim during fourth quarter | 148/746 | 0.92 (0.76 – 1.12) | 68/746 | 1.29 (1.04 – 1.59) | 140/746 | 0.96 (0.79 – 1.15)^‡^ | 390/746 | 1.01 (0.91 – 1.12) |

aOR denotes adjusted odds ratio; CDM, chronic disease management; CI, confidence interval.

* Timing of the CDM claim in the first (1-90 days), second (91-182 days), third (183-273 days), or fourth (274-365 days) quarter of the exposure period (7 to 18 months post-stroke).

† Derived using multi-level logistic regression, adjusted using inverse probability treatment weights.

‡ *P* value derived from the Wald test is <0.05, providing evidence that the coefficients are not equal across quarters.

| **Pre-event period** | | **Acute and recovery period** | | **Exposure Period** | | **Outcome Period** | |
| --- | --- | --- | --- | --- | --- | --- | --- |
| PCP persistence (regularity) and continuity  Baseline medication adherence (one year only) | |  |  | Medicare claim for a Chronic Disease Management Plan | | Medication adherence | |
| **2 years** | | **0-6 months** | | **7-18 months** | | **19-30 months** | |
|  | |  | |  | |  | |
|  | *Index event** | |  | | *^time^0* | |  |

**Figure S1. Schematic of study design**

PCP denotes Primary Care Physician.

*Defined as the first admission for stroke or transient ischaemic attack in the Australian Stroke Clinical Registry during the study period (2012-2016).

**Figure S2. Directed acyclic graph of hypothesised causal pathway**

*Baseline confounders includes demographics, stroke event details, comorbidities, pre-stroke primary and specialist care, pre-stroke medication adherence.

Solid lines indicate the pathways examined in the current analysis. Dotted lines indicate associations assumed to be incorporated in the total effect from CDM claims to medication adherence.


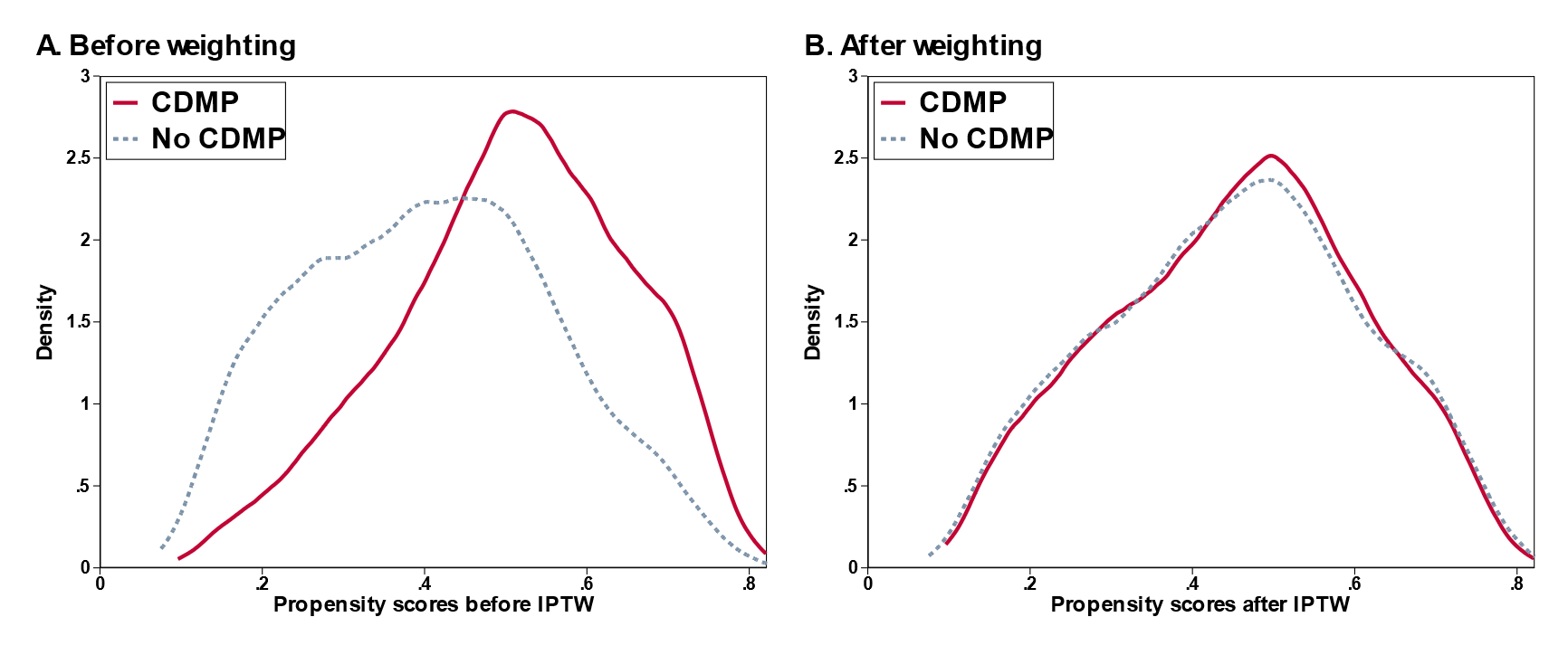


**Figure S3. Density plots of propensity scores for participants with and without a chronic disease management claim, before and after inverse probability treatment weighting**

CDMP denotes chronic disease management claim; and IPTW, inverse probability treatment weight.

**Appendix – Co-investigators and other contributors to the Australian Stroke Clinical Registry**

***Co-investigators and other contributors to the Australian Stroke Clinical Registry***

The following people are acknowledged for their contribution to collecting Hospital administrative on the patients registered in AuSCR or their participation on various governance committees:

**Steering and Management Committee**

**Craig Anderson** PhD (Royal Prince Alfred Hospital NSW, The George Institute for Global Health University of New South Wales NSW, The George Institute for Global Health at Peking University Health Science Center China); **Dominique Cadilhac** PhD (Stroke and Ageing Research, School of Clinical Sciences at Monash Health, Monash University VIC, Stroke Division, The Florey Institute of Neuroscience and Mental Health VIC, Data Custodian); **Geoffrey Donnan** MD (Stroke Division, The Florey Institute of Neuroscience and Mental Health VIC); **Rohan Grimley** MBBS (Gympie Hospital QLD, Nambour General Hospital QLD, site investigator, Sunshine Coast Clinical School, University of Queensland QLD); **Peter Hand** MBBS, MD, FRACP (Royal Melbourne Hospital VIC, site investigator)

**Steering Committee**

**Toni Aslett** BASc, GradDipBus (Stroke Foundation); **Julie Bernhardt** PhD (The Florey Institute of Neuroscience and Mental Health VIC); **Paul Bew** MPhty (The Prince Charles Hospital QLD); **Christopher Bladin** MD, MBBS, FRACP (Box Hill Hospital VIC, site investigator); **Greg Cadigan** BN (Queensland State-wide Stroke Clinical Network QLD); **Helen Castley** MBBS (Royal Hobart Hospital Tasmania); **Andrew Evans** MBBS (Hons), FRACP (Westmead Hospital NSW); **Susan Hillier** PhD (University of South Australia, SA); **Erin Lalor** PhD (Stroke Foundation VIC); **Andrew Lee** MBBS FRACP (Flinders Medical Centre, South Australia); **Richard Lindley** PhD (The George Institute for Global Health NSW); **Mark Mackay** MBBS, FRACP (Royal Children’s Hospital VIC, site investigator); **Sandra Martyn** (Health Statistics Centre Queensland Health QLD); **John McNeil** PhD (Monash University VIC); **Sandy Middleton** PhD (Nursing Research Institute, St Vincent’s Health Australia NSW, Australian Catholic University NSW); **Michael Pollack** MBBS, FAFRM (RACP), FACRM, FFPM (ANZCA), MMedSci (Clin Epi) (Hunter Stroke Service NSW); **Mark Simcocks** BSc (VIC, Consumer Representative); **Frances Simmonds** MSc(Med), (Australasian Rehabilitation Outcomes Centre NSW); **Amanda Thrift** PhD (Stroke and Ageing Research, School of Clinical Sciences at Monash Health, Monash University VIC); **Andrew Wesseldine** MBBS, FRACP (St John of God Healthcare; Department of Health WA)

**Management Committee**

**Helen Dewey** PhD (Austin Hospital VIC, Box Hill Hospital VIC, site investigator, Eastern Health Clinical School, Monash University VIC); **Steven Faux** FAFRM (RACP) (St Vincent’s Health Australia NSW); **Kelvin Hill** BAppSci (Stroke Foundation VIC); **Natasha Lannin** PhD (Faculty of Health Sciences, La Trobe University VIC, Occupational Therapy Department, Alfred Health VIC); **Christopher Levi** PhD (Acute Stroke Services, John Hunter Hospital NSW); **Christopher Price** BSocW BSc (National Stroke Foundation)

**Site Investigators**

**Lauren Arthurson** BSpPath, MHlthServMt (Echuca Regional Health VIC); **Pradeep Bambery** MD, FRCP(G), FRACP (Bundaberg Hospital QLD); **Carolyn Beltrame** RN (Div1) (Latrobe Regional Hospital VIC); **Ernie Butler** MBBS FRACP (Peninsula Health VIC); **Sean Butler** FIMLS, BM Hons, MRCP(UK), FRACP (Prince Charles Hospital QLD); **Chris Charnley** MBBS (Warrnambool Base Hospital VIC); **Ben Clissold** MBBS FRACP (University Hospital Geelong VIC); **Jo Cotterell** BPhysio (Mildura Base Hospital VIC); **Douglas Crompton** MA, PhD, MBBS, FRACP (Northern Hospital VIC); **Vanessa Crosby** Dip Physio (Albury-Wodonga Health VIC); **Carolyn De Wytt** MRCP (UK), MB BCH DUBL, FRACP (Greenslopes Private Hospital QLD); **David Douglas** MBBS, M Admin, FRACGP, FAFRM (RACP) (Ipswich Hospital QLD); **Martin Dunlop** MBBS, FACRM (Cairns Base Hospital QLD); **Paula Easton** BPhty (Hons) (Mackay Hospital QLD); **Sharan Ermel** RN (Div1) (Bendigo Health VIC); **Nisal Gange** MBBS, AMC CERT (Toowoomba Hospital QLD); **Richard Geraghty** MBBS, FRACP (Redcliffe Hospital QLD); **Kushantha Gunarathne** MBBS, MD, MMed, FRCP, FRACP (Bairnsdale Regional Health Service VIC); **Graham Hall** MBBS, FRACP (Princess Alexandra Hospital QLD); **Jonelle Hill-Uebergang** BNursing, GradDip Advanced Clinical Nursing, Advanced Dip Management (Northeast Health Wangaratta VIC); **Karen Hines** BHIM (Caboolture Hospital QLD); **Francis Hishon** RN (Redland Hospital QLD); **Joel Iedema** MBBS, FRACP (Redland Hospital QLD); **Thomas Kraemer** Approbation als Arzt, STATE EXAM MED MUNSTER, FRACP (Ballarat Health Services VIC); **Paul Laird** MBBS, FRACP (Rockhampton Hospital QLD); **Henry Ma** MBBS FRACP (Monash Medical Centre VIC); **Johanna Madden** BPhysio (Goulburn Valley Health VIC); **Graham Mahaffey** RN (Hervey Bay Hospital QLD); **Krishna Mandaleson** MBBS, FRACP, FRCP (Central Gippsland Health Service VIC); **Suzana Milosevic** MD, FRACP, AMC CERT (Logan Hospital QLD); **Peter O’Brien** MBBS, DIP RANZCOG, FRACMA, FACRRM (Warrnambool Hospital VIC); **Trisha Oxley** RN/RM, MANP (Critical Care) (Swan Hill District Health VIC); **Michaela Plante** RN (Div 1) (Rockhampton Hospital QLD); **Stephen Read** MBBS, PhD, FRACP (Royal Brisbane and Women’s Hospital QLD); **Dane Robinson** B Occ Thy (Prince Charles Hospital QLD); **Juan Rois-Gnecco** Medico Cirujano Javeriana, FAFRM (Ipswich Hospital QLD); **David Rosaia** BHlthSc, GradDipHlthSc (Bendigo Health VIC); **Kristen Rowe** BNurs, Cert NeuroSci Nurs (Austin Health VIC); **Arman Sabet** MD, FRACP, BSc (Gold Coast Hospital and Robina Hospital QLD); **Noel Saines** MBBS, FRACP (The Wesley Hospital QLD); **Eva Salud** MD, AMC CERT (Gympie Hospital QLD); **Amanda Siller** MBBS, FRACP (Queen Elizabeth II Jubilee Hospital QLD); **Christopher Staples** MD (Mater Adults QLD); **Vincent Thijs** MD FRACP PhD (Austin Hospital VIC); **Judith Walloscheck** MBA (Bendigo Health VIC); **Richard White** MD, FRCP, FRACP (Townsville Hospital QLD); **Tissa Wijeratne** (Sunshine Hospital - Western Health VIC); **Andrew Wong** MBBS, PhD (Royal Brisbane and Women’s Hospital QLD); **Lillian Wong** MBBS FRACP (Logan Hospital QLD); **Jorge Zavala** MD FRACP (Alfred Hospital VIC)

**Staff at The Florey Institute of Neuroscience and Mental Health VIC**

Robin Armstrong, Leonid Churilov, Alison Dias, Kelly Drennan, Adele Gibbs, Brenda Grabsch, Elysia Greenhill, Jen Holland, Monique Kilkenny, Joosup Kim, Charlotte Krenus, Francis Kung, Joyce Lim, Karen Moss, Kate Paice, Enna Salama, Sam Shehata, Sabrina Small, Renee Stojanovic, Steven Street, Emma Tod, Kasey Wallis, Julia Watt
